# Adjusting interlayer interactions and proton-conduction pathways of 2D covalent organic frameworks through the rotaxane structures

**DOI:** 10.1093/nsr/nwaf293

**Published:** 2025-07-30

**Authors:** Jianjian Yang, Weidong Fan, Xiaofei Wei, Ling Wei, Zhikun Wang, Wenmiao Chen, Zhelun Li, Zixi Kang, Rongming Wang, Daofeng Sun, Jianzhuang Jiang

**Affiliations:** Shandong Key Laboratory of Intelligent Energy Materials, State Key Laboratory of Heavy Oil Processing, School of Materials Science and Engineering, China University of Petroleum (East China), Qingdao 266580, China; Shandong Key Laboratory of Intelligent Energy Materials, State Key Laboratory of Heavy Oil Processing, School of Materials Science and Engineering, China University of Petroleum (East China), Qingdao 266580, China; Shandong Key Laboratory of Intelligent Energy Materials, State Key Laboratory of Heavy Oil Processing, School of Materials Science and Engineering, China University of Petroleum (East China), Qingdao 266580, China; Advanced Chemical Engineering and Energy Materials Research Center, Qingdao 266580, China; Shandong Key Laboratory of Intelligent Energy Materials, State Key Laboratory of Heavy Oil Processing, School of Materials Science and Engineering, China University of Petroleum (East China), Qingdao 266580, China; Shandong Key Laboratory of Intelligent Energy Materials, State Key Laboratory of Heavy Oil Processing, School of Materials Science and Engineering, China University of Petroleum (East China), Qingdao 266580, China; Shandong Key Laboratory of Intelligent Energy Materials, State Key Laboratory of Heavy Oil Processing, School of Materials Science and Engineering, China University of Petroleum (East China), Qingdao 266580, China; Shandong Key Laboratory of Intelligent Energy Materials, State Key Laboratory of Heavy Oil Processing, School of Materials Science and Engineering, China University of Petroleum (East China), Qingdao 266580, China; Shandong Key Laboratory of Intelligent Energy Materials, State Key Laboratory of Heavy Oil Processing, School of Materials Science and Engineering, China University of Petroleum (East China), Qingdao 266580, China; Shandong Key Laboratory of Intelligent Energy Materials, State Key Laboratory of Heavy Oil Processing, School of Materials Science and Engineering, China University of Petroleum (East China), Qingdao 266580, China; Beijing Key Laboratory for Science and Application of Functional Molecular and Crystalline Materials, Department of Chemistry, University of Science and Technology Beijing, Beijing 100083, China

**Keywords:** covalent organic framework, cyclodextrin, polyrotaxane, anhydrous proton conduction, doping

## Abstract

Covalent organic frameworks (COFs) have great potential as versatile platforms for proton conduction. However, the commonly applied 2D COFs that are easy to design and synthesize have only 1D channels for proton conduction, limiting the formation of continuous hydrogen bonds due to the anisotropy between their crystalline grains. Herein, we report a strategy to construct 3D channels in 2D COFs by using rotaxane structures and eliminate the strong interlayer π–π interactions, facilitating the formation of smooth 3D proton-transfer pathways during guest doping. The presence of interlocking α-cyclodextrin (CD) molecules in a rotaxane-based COF (CD-TpAzo) significantly diminishes the stacking energy between the 2D layers from 154.2 to 55.2 kJ mol^−1^, resulting in easier H_3_PO_4_ doping into its 3D channels and interlayers. As a result, CD-TpAzo@H_3_PO_4_-10 exhibits an eight times shorter H^+^ spin-lattice relaxation time than TpAzo@H_3_PO_4_-10. At 150°C, the anhydrous proton conductivity of CD-TpAzo@H_3_PO_4_-18 reaches 0.78 S cm^−1^, which is even higher than that of pure H_3_PO_4_ (0.47 S cm^−1^) under the same conditions.

## INTRODUCTION

Proton conductors are a class of solid conductive materials that carry charges through a proton medium [1]. These materials have been studied extensively in recent years due to their potential applications in fuel cells [2], humidity and gas sensors [3], capacitors [4] and other energy-related technologies. The primary focus of proton conductor research lies in revealing their structure–function relationship to develop materials with high proton conductivity, low cost and good stability. As the key component for fuel cells, proton conductors significantly affect their output performance and life [5–8]. As a result, researchers have explored various proton-conducting materials, including polymers, ceramics and metal oxides [9–14]. Among them, nafion—a perfluorosulfonic acid polymeric membrane developed by DuPont—has attracted much attention due to its high proton conductivity and mechanical stability at room temperature [15]. However, its narrow operating temperature (0–80°C) [16], high methanol permeability [17] and difficulty in synthetic processing [18] restrict its widespread application. Additionally, the amorphous structure of polymers makes it difficult to elucidate the relationship between structure and performance, and further improve its functionality accordingly [19]. Developing new proton-conducting materials with defined structures is therefore one of the promising development directions for proton exchange membrane fuel cells [20–22].

Covalent organic frameworks (COFs) are a class of 2D or 3D crystalline porous materials made by periodically linked organic molecular building blocks via covalent bonds [23–26]. Their highly ordered pore structure and high specific surface area provide a favorable environment for efficient proton transport [27–30], therefore acting as promising versatile platforms for proton conduction. In addition, covalent bonds between organic building blocks afford a strong and stable framework, making them ideal for proton-conduction applications [31–35]. However, most COFs that are easy to design and synthesize possess 2D layered structures with highly rigid frameworks and strong interlayer π–π interactions [36]. As a consequence, protons can only conduct through 1D pore channels, making it difficult to form continuous hydrogen-bond networks over bulk COF materials due to the anisotropy between COF crystalline grains [37]. In previous work, we demonstrated that increasing the number of hydrophilic groups in 2D COF channels can enable doped H_3_PO_4_ to enter their interlayers to construct smooth proton-transfer pathways. However, overcoming strong interlayer π–π interactions remains difficult [38].

Mechanically interlocked molecules (MIMs) have been designed and synthesized for various materials across diverse fields [39–42]. Among the MIMs, the high structural freedom and mobility of rotaxane units in rotaxane-based mechanically interlocked polymers (MIPs) endow them with unique properties, including mechanical robustness, adaptability and responsiveness [43,44]. Inspired by the characteristics of MIPs, herein we develop a novel strategy that introduces rotaxane structures into the framework of 2D COFs to form 3D channels, weakening the interactions between 2D layers and facilitating the entry of small guest molecules into their interlayers, thereby expanding their proton-conduction pathways. The rotaxane-based COF (RCOF, CD-TpAzo) was prepared by the amine-aldehyde condensation of pseudorotaxane assembled by α-cyclodextrin (CD) and 4,4′-azodianiline (Azo) with 1,3,5-triformylphroglucinol (Tp). In comparison with the 154.2 kJ mol^−1^ of the π–π stacking interaction in TpAzo prepared by condensation between Tp and Azo, theoretical calculation reveals the significantly reduced stacking energy between 2D layers (55.2 kJ mol^−1^) due to the rotaxane structures in CD-TpAzo. Powder X-ray diffraction (PXRD), atomic force microscopy (AFM) and solid-state nuclear magnetic resonance (ssNMR) studies demonstrate the facile doping of H_3_PO_4_ into not only the 3D channels, but also the interlayer spaces of CD-TpAzo to afford CD-TpAzo-x (x represents the doping volume of H_3_PO_4_) because of the reduced interlayer interactions. Owing to the formation of the more continuous 3D proton-conduction pathways, the H^+^ spin-lattice relaxation of CD-TpAzo@H_3_PO_4_-10 is eight times faster than that of TpAzo@H_3_PO_4_-10. At 150°C, the anhydrous proton conductivity of CD-TpAzo@H_3_PO_4_-18 reaches 0.78 S cm^−1^, exceeding the corresponding value for pure H_3_PO_4_ under the same conditions (0.47 S cm^−1^) [45]. In addition, when a 5% CD-TpAzo@H_3_PO_4_-18/nafion composite membrane is used as the proton exchange membrane of an H_2_/O_2_ single cell, the maximum power density reaches 248 mW cm^−2^ at 70°C and under anhydrous conditions—38% higher than the recast nafion membrane under the same conditions—together with high stability over a 12-hour period without obvious power loss.

## RESULTS AND DISCUSSION

### Synthesis, structure and characterization

The pseudorotaxane CD-Azo was prepared by the self-assembly of α-cyclodextrin and Azo with a molar ratio of 1:1 in an aqueous solution with pH 10 adjusted by using NaOH. As shown in Fig. S1, the ^1^H NMR spectra show that the proton chemical shifts of the two CH and NH groups in Azo occur at 7.51, 6.63 and 5.69 ppm, but, in CD-Azo, they shift significantly towards higher fields and appear at 7.64, 6.75 and 5.74 ppm, respectively, due to the shielding effect of cyclodextrin, indicating that the pseudorotaxane was successfully assembled. Meanwhile, this is also demonstrated by the slight blue shifts of the N=N stretching vibration of Azo and the C–O–C stretching vibrations of cyclodextrin from 1592 and 1029 cm^−1^ to 1593 and 1031 cm^−1^, respectively, in the Fourier-transform infrared (FT–IR) spectra of Azo and CD-Azo (Fig. S2).

**Figure 1.**
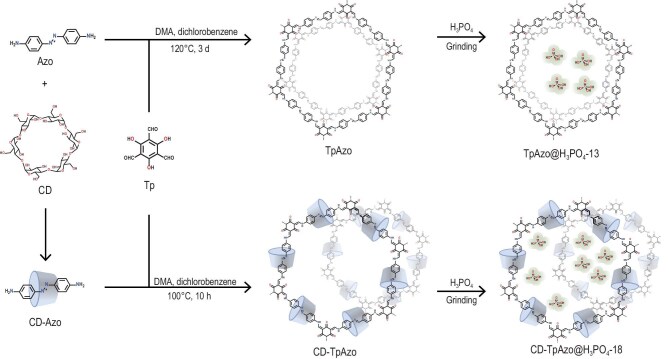
Synthesis of CD-TpAzo@H_3_PO_4_-13 and CD-TpAzo@H_3_PO_4_-18.

**Figure 2.**
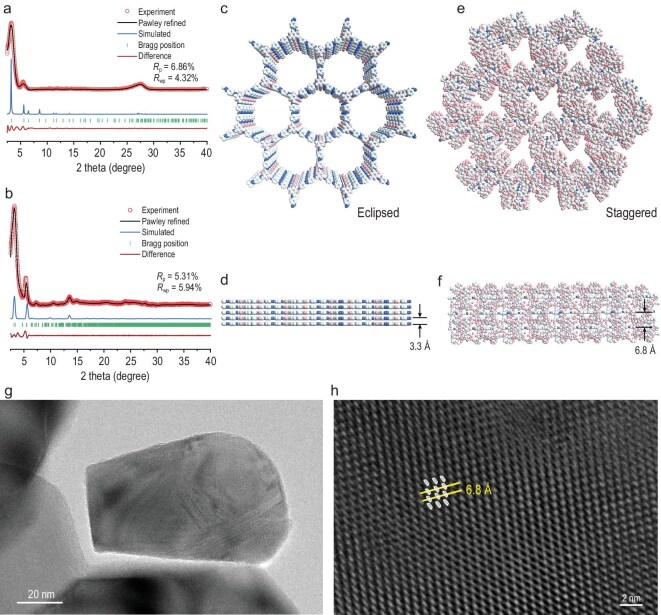
PXRD patterns of (a) TpAzo and (b) CD-TpAzo; eclipsed stacking models of (c) front view and (d) side view for TpAzo; staggered stacking models of (e) front view and (f) side view for CD-TpAzo; (g, h) side HRTEM image of CD-TpAzo.

The rotaxane-based CD-TpAzo was synthesized by the amine-aldehyde condensation reaction of CD-Azo and Tp in a mixture of dimethylacetamide (DMA) and *o*-dichlorobenzene (v/v = 1:1) at 100°C for 10 hours (Fig. 1). For comparison, the conventional COF (TpAzo) composed of Tp and Azo was also synthesized via similar procedures [46]. The crystallinity of COFs was evaluated by using PXRD analysis (*λ* = 0.15 406 Å). Similarly to the reported reference, the PXRD pattern of TpAzo shows a strong peak at 2*θ* = 3.2° and a weak peak at 27°, corresponding to 100 and 001 plane reflections, respectively, with a π–π stacking distance of 3.3 Å between the 2D layers (Fig. 2a, c and d). When CD was assembled into the COF framework, the PXRD pattern of CD-TpAzo exhibited five peaks at 2*θ* = 3.2°, 5.5°, 7.2°, 10.5° and 13.6°, and the distance between the 2D layers was calculated to be 6.8 Å, which is significantly larger than that in TpAzo (3.3 Å) (Fig. 2b, e and f). This distance is no longer determined by the π–π interaction within the COF layers, while the hydrogen bonds between cyclodextrins are at work. The side high-resolution transmission electron microscopy (HRTEM) image of CD-TpAzo confirms this point (Fig. 2g and h), in which lattice fringes with a spacing of 6.8 Å can be observed. The possible 2D model with an eclipsed structure in the hexagonal space group (*P*6/*m*) and a staggered structure in the *P*1 space group was constructed by using Materials Studio 2018 software. The experimental PXRD pattern closely matches the simulated pattern of the eclipsed stacking model. To find the unit cell parameters, Pawley refinement was performed on both COFs. The unit cell values were found to be *a* = *b* = 31.5 Å, *c* = 3.3 Å for TpAzo and *a* = *b* = 31.5 Å, *c* = 13.6 Å for CD-TpAzo. The intensity ratio of the staggered form matches the PXRD of CD-TpAzo (Tables S6 and S7).

**Figure 3.**
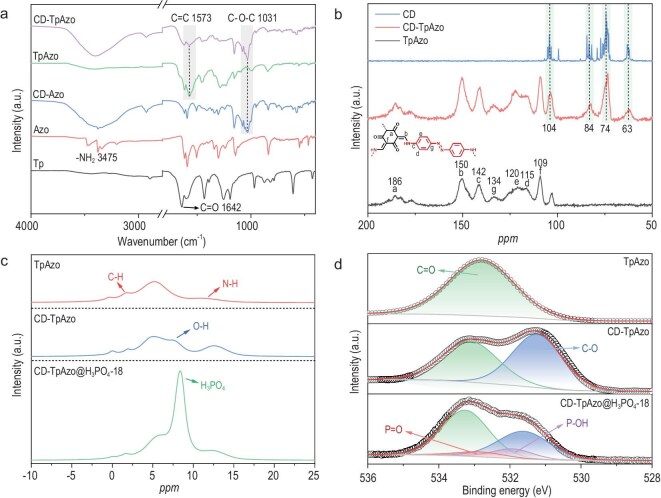
(a) FT–IR spectra of Tp, Azo, CD-Azo, TpAzo and CD-TpAzo; (b) solid-state ^13^C NMR spectrum of CD, TpAzo and CD-TpAzo; (c) comparison of ^1^H single-pulse spectra of TpAzo, CD-TpAzo and CD-TpAzo@H_3_PO_4_-18; (d) high-resolution XPS spectra of O 1s for TpAzo, CD-TpAzo and CD-TpAzo@H_3_PO_4_-18.

As shown in Fig. 3a, the appearance of the C=C stretching vibrations at 1573 cm^−1^ and the disappearance of the C=O stretching vibrations at 1642 cm^−1^ demonstrate the occurrence of the Schiff–base coupling reaction in the FT–IR spectra of TpAzo and CD-TpAzo, and an additional signal at 1031 cm^−1^ corresponding to the C–O–C stretching vibrations of CD is observed in the FT–IR spectrum of CD-TpAzo, indicating the structural integrity of the rotaxane-based host–guest covalent organic framework. This is further confirmed by the solid-state ^13^C nuclear magnetic resonance (^13^C NMR) spectra (Fig. 3b). It can be seen that the peaks of TpAzo corresponding to the C=O, C=C and C–N bonds appear at 186, 150, 142, 133, 120 and 109 ppm, respectively, while the signals of CD-TpAzo not only show the characteristic peaks of CD at 63, 74, 84 and 104 ppm, respectively, but also the characteristic peaks of the TpAzo framework that are slightly shifted due to the shielding effect of the CD macrocycle on the host framework (Fig. S3). Meanwhile, the solid-state ^1^H NMR spectrum of CD-TpAzo clearly shows the presence of –OH, further demonstrating the successful synthesis of the rotaxane-based COF (Fig. 3c). The results of elemental analysis also confirmed this point (Table S1).

The above conclusion was also supported by X-ray photoelectron spectroscopy (XPS) and N_2_ adsorption–desorption experiments. The XPS spectra of TpAzo and CD-TpAzo display three evident peaks corresponding to C 1s, N 1s and O 1s, respectively (Fig. S4). Compared with the O 1s high-resolution XPS spectrum of TpAzo, the O atom binding energy of the C=O bonds shifts from 532.8 to 533.1 eV and a new peak appears at 531.2 eV attributed to the C–O bonds of CD for CD-TpAzo (Fig. 3d). These results further confirm the existence of CD molecules and their supramolecular interactions with the host framework of CD-TpAzo. The porosity of TpAzo and CD-TpAzo was investigated by measuring the N_2_ adsorption–desorption of fully activated samples at 77 K under 1 atm. Their adsorption curves exhibit type-Ⅱ isotherms due to the presence of crystalline imperfect phases (Fig. S5a–d). The N_2_ uptakes of TpAzo and CD-TpAzo are 533.0 and 396.8 cm^3^ g^−1^, with Brunauer Emmett-Teller (BET) surface areas 383.8 and 280.7 m^2^ g^−1^, respectively. As expected, CD-TpAzo has smaller porosity and surface area due to the mechanical insertion of CD into the framework and the pore size (15.0 Å) of CD-TpAzo is also smaller than that of TpAzo (25.5 Å). The adsorption and desorption tests of CO_2_ at 196 K showed corresponding values of 15.6 and 26.2 Å, respectively (Fig. S5e and f). They are basically consistent with the calculated values (16.5 and 27.3 Å) based on density functional theory (DFT), further confirming the correctness of the simulated CD-TpAzo structure. The adsorption and desorption test results of carbon dioxide at 196 K also confirmed this result.

### Stability test

To determine the effect of introducing mechanically intercalated guest CD molecules on the structure stability of the RCOF host framework, 50 mg of TpAzo and CD-TpAzo were treated under different stringent conditions. It can be seen that there is no change in the PXRD patterns of both TpAzo and CD-TpAzo after being heated in an oven for 24 hours up to 170°C, indicating their excellent thermal stability (Fig. S6a and c). In fact, thermogravimetric analyses show that TpAzo and CD-TpAzo have almost identical thermal stability up to 300°C (Fig. S7). When 50 mg of TpAzo and CD-TpAzo were placed in 20 mL of different concentrations of H_3_PO_4_ for 3 days, the PXRD patterns show that the intensity of TpAzo changes slightly in 6 M H_3_PO_4_, but CD-TpAzo does not change, indicating that the existence of guest CD molecules makes CD-TpAzo have better chemical stability (Fig. S6b and d). These results display that CD-TpAzo is an attractive candidate as an acid-doped high-temperature anhydrous proton conductor.

### H_3_PO_4_ doping

TpAzo@H_3_PO_4_-x and CD-TpAzo@H_3_PO_4_-x (x represents the volume of H_3_PO_4_) were obtained by doping 10 mg of as-synthesized TpAzo and CD-TpAzo with different amounts of H_3_PO_4_ and drying at 120°C under a vacuum for 12 hours, and the accurate H_3_PO_4_ content was tested by using inductively coupled plasma mass spectra (ICP–MS). The results show that the maximum H_3_PO_4_ doping capacities of TpAzo and CD-TpAzo are 13 and 18 µL, respectively, denoted as TpAzo@H_3_PO_4_-13 and CD-TpAzo@H_3_PO_4_-18. ICP tests show that the maximum H_3_PO_4_ doping capacity of TpAzo is 2.40 mg/mg, which is slightly higher than the theoretical value of 2.28 mg/mg calculated according to its porosity owing to the surface adsorption. However, the maximum H_3_PO_4_ doping amount of CD-TpAzo is 3.34 mg/mg—1.6 times the theoretical value of 2.03 mg/mg—because H_3_PO_4_ not only enters the 3D pores of CD-TpAzo, but also enters its interlayers. This can be demonstrated by a series of studies. As shown in Fig. S8, the P=O stretching peak at 1005 cm^−1^ appears in the FT–IR spectra of TpAzo@H_3_PO_4_-13 and CD-TpAzo@H_3_PO_4_-18, indicating the successful doping of H_3_PO_4_. Different from the FT–IR spectrum of TpAzo, CD-TpAzo displays an obvious peak at 3400 cm^−1^ corresponding to the –OH stretching vibrations, which, however, almost disappeared in the FT–IR spectrum of CD-TpAzo@H_3_PO_4_-18, meaning that the doped H_3_PO_4_ has a strong interaction with the –OH of the CD. However, after being washed with water, the FT–IR spectrum is almost restored to its original shape (Fig. S9), indicating that the 2D layer structure of CD-TpAzo can remain stable during grinding with H_3_PO_4_. The N_2_ adsorption tests demonstrate that the adsorption capacities of TpAzo@H_3_PO_4_-13 and CD-TpAzo@H_3_PO_4_-18 decrease to 29.4 and 27.6 cm^3^ g^−1^, respectively, suggesting their filled pores with H_3_PO_4_ (Fig. S5a and S5b). After being washed with water, their adsorption capacities recover to 282.5 and 224.5 cm^3^ g^−1^, respectively, indicating that the H_3_PO_4_ in their pores can be washed away. More importantly, compared with the PXRD pattern of TpAzo, the peak intensity of TpAzo@H_3_PO_4_-13 only decreases at 2*θ* = 3.2° due to its pores being filled with H_3_PO_4_ and returns to the same as before doping after washing with water, proving that the ordered structure of TpAzo still remains after doping (Fig. S10a). However, the PXRD curve of CD-TpAzo completely disappears after doping with H_3_PO_4_, indicating the formation of a disordered structure after H_3_PO_4_ loading, but, as expected, the PXRD pattern of CD-TpAzo completely returns after washing with water, showing the restoration of ordered structures (Fig. S10b). This indicates that the 2D layers of CD-TpAzo are not destroyed during H_3_PO_4_ doping, but H_3_PO_4_ enters its interlayers, causing sliding between the 2D layers, resulting in structural disorder. After the H_3_PO_4_ was washed away, hydrogen-bonding interactions between the –OH groups of CDs on the 2D layers reoriented the ordered structure of CD-TpAzo. This is also why CD-TpAzo has a smaller pore volume but can load more H_3_PO_4_ than TpAzo. This point can also be supported by using scanning electron microscopy (SEM) testing. As shown in Fig. S11a and b, both TpAzo and CD-TpAzo have nanofiber-like morphologies. As the H_3_PO_4_ doping amount increases, the crystal morphology of TpAzo does not show any significant change and is only adhered together by the H_3_PO_4_ on its surface (Fig. S12). However, the crystal morphology of CD-TpAzo gradually transforms into a layered structure (Fig. S13), clearly indicating the interlayer insertion of H_3_PO_4_. The corresponding mappings further demonstrate that H_3_PO_4_ can be effectively doped into TpAzo and CD-TpAzo (Figs S14 and S15).

To understand the state of H_3_PO_4_ in CD-TpAzo@H_3_PO_4_-18, the liquid-state ^31^P NMR spectrum of 85% H_3_PO_4_ used in this work and the solid-state ^31^P NMR spectrum of CD-TpAzo@H_3_PO_4_-18 were obtained for comparative study (Fig. S16). The liquid-state spectrum of 85% H_3_PO_4_ exhibits a peak at 0 ppm, but the solid-state spectrum of CD-TpAzo@H_3_PO_4_-18 shows a strong peak at 0.38 ppm and a tiny peak at −12.09 ppm, which can be attributed to H_3_PO_4_ and H₄P₂O₇. Integration indicates that ∼0.8% of the H_3_PO_4_ dehydrated to form H₄P₂O₇ during the vacuum drying. In addition, XPS studies show two new peaks corresponding to P 2s and P 2p, besides the peaks attributed to C 1s, N 1s and O 1s in the survey spectrum of CD-TpAzo@H_3_PO_4_-18 (Figs S17 and S18). The high-resolution O 1s XPS spectrum of CD-TpAzo@H_3_PO_4_-18 can be curve-fitted into four signals at 532.9, 531.9, 533.3 and 531.6 eV corresponding to P=O, P–OH, C=O and C–O, respectively (Fig. 3d). Meanwhile, the N 1s spectrum of CD-TpAzo can be deconvoluted into two signals attributed to C–N–C and C–N=N at 399.6 and 400.1 eV, and the corresponding signals slightly shift to 399.8 and 400.3 eV for CD-TpAzo@H_3_PO_4_-18 due to the protonation. These results further confirm the successful doping of H_3_PO_4_ (Fig. S19).

**Figure 4.**
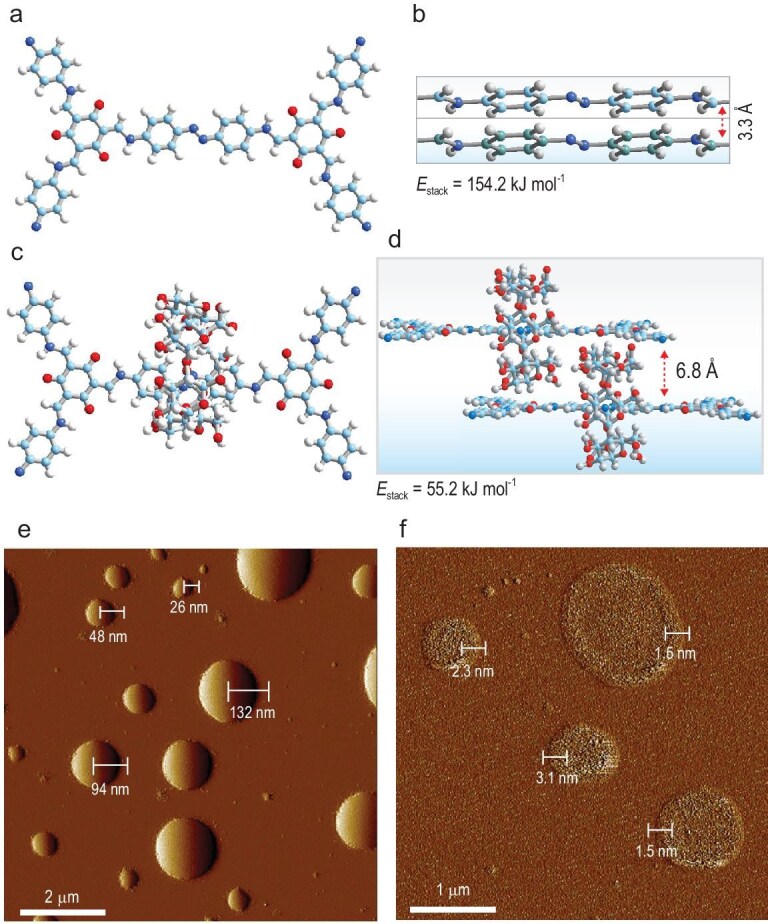
Stacked 2D layers of TpAzo ((a) top view and (b) side view) and CD-TpAzo ((c) top view and (d) side view), and the AFM thickness images of (e) TpAzo@H_3_PO_4_-13 and (f) CD-TpAzo@H_3_PO_4_-18 after ultrasound in water.

To investigate the influence of rotaxane structures on the interlayer interactions of 2D RCOF, the π–π stacking energy (*E*_stack_) of the unit cell bilayers was calculated by using DFT. The results show that the *E*_stack_ of TpAzo is 154.2 kJ mol^−1^, but the *E*_stack_ of CD-TpAzo is significantly reduced to 55.2 kJ mol^−1^, indicating that the introduction of CD greatly weakens the interlayer interactions of 2D RCOF (Fig. 4a–d). Meanwhile, the water contact angles of TpAzo and CD-TpAzo were tested by using the JC2000 contact angle measuring instrument (Fig. S20). The results show that the water contact angle of TpAzo is 75°, but that of CD-TpAzo drops significantly to 24° after introducing the interlocking CD on the framework of TpAzo. The weakening of interlayer interactions and the enhancement of the surface hydrophilicity of CD-TpAzo are conducive to the entry of H_3_PO_4_ molecules into its interlayers to construct smoother 3D proton-transfer pathways. To further confirm whether H_3_PO_4_ molecules can enter the interlayers of CD-TpAzo, both TpAzo and CD-TpAzo were exfoliated ultrasonically in 5% dilute H_3_PO_4_ solution for 30 minutes and observed under AFM. It can been seen that TpAzo can only be dispersed into nanospheres with different particle sizes, but CD-TpAzo can be exfoliated into nanosheets with different thicknesses, such as 1.5, 2.3 and 3.1 nm, which correspond to the thicknesses of single-layer [47], bilayer and trilayer polyrotaxane COF structures (Fig. 4e and f, and Fig. S21). This result provides good evidence for the fact that H_3_PO_4_ molecules can enter the interlayer of CD-TpAzo.

### Proton conductivity

Based on the design of a polyrotaxane structure, we used CD-TpAzo as an anhydrous proton-conducting platform by doping H_3_PO_4_ into its channels and interlayers via the hydrogen-bonding interactions of H_3_PO_4_ with –OH and –N=N– groups on the CD and TpAzo frameworks. All samples were compressed into small pellets with a diameter of 3 mm and a thickness of ∼0.3 mm (Fig. S22). The anhydrous proton conductivities were studied by using a 1260A Impedance/Gain-Phase Analyzer from 10 MHz to 0.1 Hz with an input voltage of 200 mV in the temperature range of 70–150°C. As shown in Fig. S23, the anhydrous proton conductivities of the as-synthesized TpAzo and CD-TpAzo are only 2.39 × 10^−7^ and 3.15 × 10^−7^ S cm^−1^, respectively, even at 150°C. With an increase in the H_3_PO_4_ doping amount, the proton conductivity gradually increases (Figs S24 and S25). At 150°C, the proton conductivities of TpAzo@H_3_PO_4_-5, TpAzo@H_3_PO_4_-10 and TpAzo@H_3_PO_4_-13 are 1.41 × 10^−5^, 3.72 × 10^−4^ and 5.74 × 10^−3^ S cm^−1^, respectively (Table S2), but the values of CD-TpAzo@H_3_PO_4_-5, CD-TpAzo@H_3_PO_4_-10, CD-TpAzo@H_3_PO_4_-15 and CD-TpAzo@H_3_PO_4_-18 are 2.44 × 10^−4^, 2.83 × 10^−3^, 6.15 × 10^−2^ and 0.78 S cm^−1^, respectively (Table S3). It can be seen that, when doped with the same amount of H_3_PO_4_, the proton conductivity of CD-TpAzo@H_3_PO_4_-x is about an order of magnitude higher than that of TpAzo@H_3_PO_4_-x. Obviously, this is because H_3_PO_4_ can enter the 3D pores and interlayers of CD-TpAzo to construct the continuous 3D proton-transfer pathway as demonstrated by the above FT–IR, PXRD, SEM, HRTEM and AFM studies.

**Figure 5.**
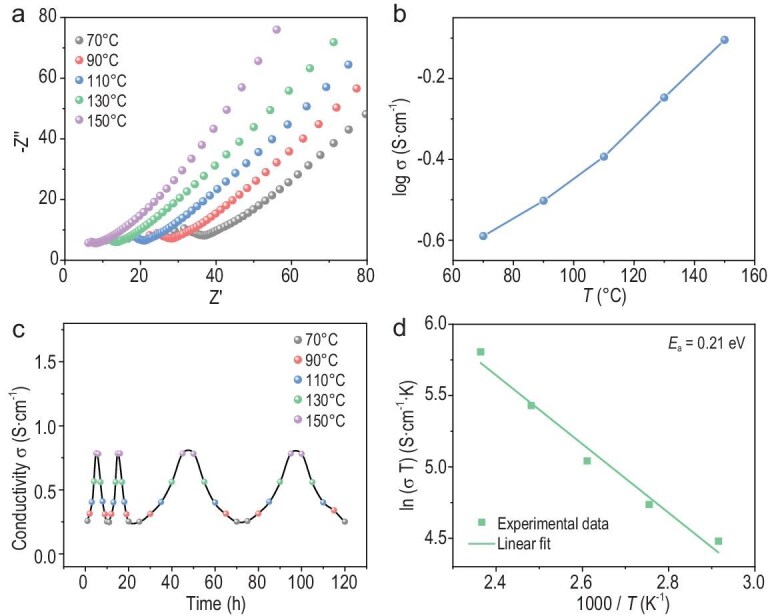
(a) Nyquist plot, (b) log-scaled proton conductivities, (c) cyclic proton conductivities and (d) Arrhenius plots of CD-TpAzo@H_3_PO_4_-18 under various temperatures.

Moreover, the temperature-dependent impedance studies display that the temperature has a significant influence on their proton conductivities. At 70°C, the conductivity of TpAzo@H_3_PO_4_-13 is 1.29 × 10^−3^ S cm^−1^, but, as the temperature increases to 150°C, its conductivity reaches 5.74 × 10^−3^ S cm^−1^. Notably, CD-TpAzo@H_3_PO_4_-18 exhibits an ultra-high conductivity of 0.26 S cm^−1^ even at 70°C, which increases to 0.78 S cm^−1^ at 150°C (Fig. 5a and b, and Table S3). This value is higher than that of pure H_3_PO_4_ (0.47 S cm^−1^) under the same condition, indicating that the hydrogen-bonding networks formed by H_3_PO_4_ in CD-TpAzo@H_3_PO_4_-18 are more orderly than those of pure H_3_PO_4_. The long-term durability tests and cyclic proton conductivity tests show that the proton conductivity of CD-TpAzo@H_3_PO_4_-18 maintains no major change over 120 hours (Fig. 5c and Fig. S26). These results demonstrate that the ordered layered structures of 2D RCOF play a crucial role in constructing smoother proton-transfer pathways for doped guest molecules.

**Figure 6.**
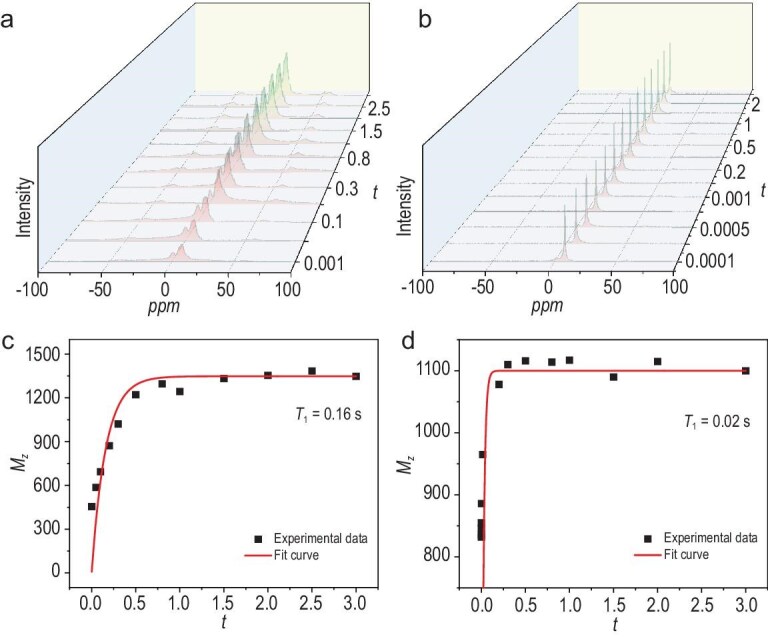
Spin-lattice relaxation of H^+^ in (a) TpAzo@H_3_PO_4_-10 and (b) CD-TpAzo@H_3_PO_4_-10, and the relaxation time fitting diagram of H^+^ in (c) TpAzo@H_3_PO_4_-10 and (d) CD-TpAzo@H_3_PO_4_-10.

The proton-conduction mechanism of CD-TpAzo@H_3_PO_4_-18 was also studied through the activation energy (*E*_a_) of proton transfer, which was obtained by fitting the temperature-dependent proton conductivities based on the Arrhenius equation. As shown in Fig. S27, the *E*_a_ values of TpAzo@H_3_PO_4_-5, TpAzo@H_3_PO_4_-10 and TpAzo@H_3_PO_4_-13 are 0.46, 0.38 and 0.26 eV, respectively. This indicates that the formation of ordered hydrogen bonds is accompanied by an increase in H_3_PO_4_ doping and the proton transfers of TpAzo@H_3_PO_4_-x gradually change from a vehicle mechanism (*E*_a_ > 0.4 eV) to a Grotthuss mechanism (*E*_a_ < 0.4 eV). Correspondingly, the *E*_a_ values of CD-TpAzo@H_3_PO_4_-5, CD-TpAzo@H_3_PO_4_-10, CD-TpAzo@H_3_PO_4_-15 and CD-TpAzo@H_3_PO_4_-18 are 0.31, 0.29, 0.24 and 0.21 eV, respectively, showing that the proton transfer of CD-TpAzo@H_3_PO_4_-x has smaller activation than that of TpAzo@H_3_PO_4_-x and always adopts the Grotthuss mechanism (Fig. 5d and Fig. S28). This further confirms that CD-TpAzo is more likely to form continuous and ordered hydrogen-bonding networks after doping with H_3_PO_4_. To gain a deeper understanding of the proton-conduction mechanism of CD-TpAzo@H_3_PO_4_-x, the proton motility of TpAzo@H_3_PO_4_-10 and CD-TpAzo@H_3_PO_4_-10 was studied by using solid-state ^1^H NMR spectra (Fig. 6). It can be clearly seen that the motility of H^+^ in CD-TpAzo@H_3_PO_4_-10 is significantly higher than that in TpAzo@H_3_PO_4_-10, as shown by the spin-lattice relaxation time *T*_1_. After excitation, the signal intensity at 8.4 ppm corresponding to the H^+^ of H_3_PO_4_ rapidly increases and stabilizes, indicating that H^+^ returns to the ground state (Fig. 6a and b). The data fitting displays *T*_1_ = 0.16 s for TpAzo@H_3_PO_4_-10 and *T*_1_ = 0.02 s for CD-TpAzo@H_3_PO_4_-10 (Fig. 6c and d), meaning that the motility of H^+^ in CD-TpAzo@H_3_PO_4_-10 is eight times faster than that in TpAzo@H_3_PO_4_-10. Molecular dynamics simulations show that the diffusion coefficient of the H^+^ in CD-TpAzo@H_3_PO_4_ (*D* = 3.52 × 10^−8^ m^2^ s^−1^) is 11.9 times that in TpAzo@H_3_PO_4_ (*D* = 2.96 × 10^−9^ m^2^ s^−1^) at 150°C (Fig. S29a), which is consistent with the room-temperature test results of the solid-state ^1^H NMR spectra. The length of the hydrogen bond formed by –OH with H_3_PO_4_/H_2_PO_4_^−^ in CD-TpAzo@H_3_PO_4_ is mostly distributed at 1.4 Å, whereas the lengths of the hydrogen bonds formed by –NH– and –C=O with H_3_PO_4_/H_2_PO_4_^−^ on the TpAzo@H_3_PO_4_ framework are mostly distributed at 1.6 Å (Fig. S29b). This indicates that –OH is able to immobilize H_3_PO_4_/H_2_PO_4_^−^ and form continuous and ordered hydrogen-bonding networks. This result further proves that CD-TpAzo@H_3_PO_4_-x possesses smoother proton-conduction pathways because H_3_PO_4_ molecules form more continuous and ordered hydrogen-bonding networks due to the elimination of the anisotropy of hydrogen-bonding channels between nanosheets and the confinement effect of the 2D layers on it in CD-TpAzo. As illustrated in Fig. S30, for TpAzo with 1D channels, it is difficult to form a long-range ordered hydrogen-bonding network through the H_3_PO_4_ molecules in the channels due to the disordered arrangement between the particles. However, for CD-TpAzo, due to the ability of H_3_PO_4_ molecules to enter its 3D pores and 2D interlayers, H_3_PO_4_ molecules can easily form a continuous hydrogen-bonding network even if the nanosheets are disorderly arranged.

To evaluate the practical application effect of CD-TpAzo@H_3_PO_4_-18, a 5% CD-TpAzo@H_3_PO_4_-18/nafion composite membrane was prepared by mixing CD-TpAzo@H_3_PO_4_-18 and nafion, and the recast nafion membrane was also obtained for comparison. H_2_ permeability testing shows that the gas permeability of 5% CD-TpAzo@H_3_PO_4_-18/nafion was 9.12 ± 0.85 barrer, which is only slightly higher than the value of the recast nafion membrane (8.06 ± 0.89 barrer) (Table S4), indicating that the doping of a small amount of CD-TpAzo@H_3_PO_4_-18 has little effect on the gas permeation performance of nafion membranes. Subsequently, two types of membranes were assembled separately into membrane electrode assemblies (MEAs) as a solid-state electrolyte within real H_2_/O_2_ single fuel cells for performance testing (Fig. S31). At 70°C and under anhydrous conditions, the open-cell voltage of the MEAs prepared by a 5% CD-TpAzo@H_3_PO_4_-18/nafion composite membrane is 0.878 V, which is slightly lower than that of the MEAs made with the recast nafion membrane (0.882 V), but the peak current density and power density of the 5% CD-TpAzo@H_3_PO_4_-18/nafion composite membrane are 549 mA cm^−2^ and 248 mW cm^−2^, which are 19% and 38% higher than those of the recast nafion membrane (460 mA cm^−2^ and 180 mW cm^−2^), respectively (Fig. S32a), indicating that a moderate incorporation of CD-TpAzo@H_3_PO_4_-18 can significantly improve the performance of the nafion membrane. Meanwhile, the long-term discharge durability of the composite membrane was also studied for 12 hours at 70°C and under anhydrous conditions, showing that the performance of the membrane did not change significantly after continuous discharge (Fig. S32b). As shown in Fig. S33, the tensile strengths of the recast nafion membrane and 5% CD-TpAzo@H_3_PO_4_-18/nafion composite membranes were 25.36 and 25.21 MPa, respectively. The incorporation of 5% CD-TpAzo@H_3_PO_4_-18 did not degrade the mechanical properties of the composite membranes, which indicated that there was good compatibility between 5% CD-TpAzo@H_3_PO_4_-18 and Nafion. These results prove the application potential of CD-TpAzo@H_3_PO_4_-18 in composite membranes.

## CONCLUSIONS

In summary, we designed and developed a rotaxane-based covalent organic framework, CD-TpAzo, as the platform for anhydrous proton conduction. The α-cyclodextrin molecules interlocked on the framework of RCOF result in the formation of 3D channels and weaken the interlayer interactions, making it easier for H_3_PO_4_ to insert into its interlayers and expanding the proton-transport pathways from 1D to 3D, which is conducive to the formation of long-range ordered hydrogen-bonding networks between anisotropic arranged RCOF nanosheets. Meanwhile, the confinement effect of the 2D layers in RCOF also helps H_3_PO_4_ to form more ordered hydrogen-bonding networks between the layers. As a result, CD-TpAzo@H_3_PO_4_-18 has an ultra-high anhydrous proton conductivity of 0.78 S cm^−1^ at 150°C. This work not only provides a new idea for affording 2D COFs with 3D channels as proton conductors, but also offers a new method for preparing 2D COFs that are prone to interlayer delamination.

## Supplementary Material

nwaf293_Supplemental_File
