# Supplementary material for: Adjusting interlayer interactions and proton-conduction pathways of 2D covalent organic frameworks through the rotaxane structures
Source: Natl Sci Rev. 2025 Jul 30;12(9):nwaf293. doi: 10.1093/nsr/nwaf293 (PMC12416279; doi:10.1093/nsr/nwaf293)
Supplement: nwaf293_Supplemental_File [file nwaf293_supplemental_file.pdf]

Supporting Information

Adjusting interlayer interactions and proton conduction pathways of 2D covalent organic frameworks through the rotaxane structures

Jianjian Yang[1], Weidong Fan[1,*], Xiaofei Wei[1], Ling Wei[2], Zhikun Wang[1], Wenmiao Chen[1], Zhelun Li[1], Zixi Kang[1], Rongming Wang[1,*], Daofeng Sun[1] and Jianzhuang Jiang[3,*]

[1]Shandong Key Laboratory of Intelligent Energy Materials, State Key Laboratory of Heavy Oil Processing, School of Materials Science and Engineering, China University of Petroleum (East China), Qingdao Shandong 266580, China

[2]Advanced Chemical Engineering and Energy Materials Research Center, Qingdao Shandong 266580, China

[3]Beijing Key Laboratory for Science and Application of Functional Molecular and Crystalline Materials, Department of Chemistry, University of Science and Technology Beijing, Beijing 100083, China

***Corresponding authors.**

E-mail: jianzhuang@ustb.edu.cn, rmwang@upc.edu.cn, wdfan@upc.edu.cn

Table of Contents

Section		Page No
**S-1**	**General Information**	3
**S-2**	**Experimental Procedures**	7
**S-3**	**Preparation of precursors (CD-Azo)**	12
**S-4**	**Characterization of the pristine COFs**	13
**S-5**	**Confirmation and characterization of the phosphoric acid doped COF samples**	16
**S-6**	**Proton conductivity measurement results**	24
**S-7**	**Supporting tables**	30
**S-8**	**Supporting references**	42

Section S-1: General Information

Materials and Physical Measurements.

All the chemical reagents were commercially available and used without further purification. 1,3,5-triformylphloroglucinol (99.9%), p-Nitroaniline (99.9%) and α-cyclodextrin (98%) were bought from Sigma Aldrich (China). Sodium bisulfite ($NaHSO_3$) (98%), sodium nitrite ($NaNO_2$) (98%), sodium sulfide nonahydrate (Na_2S $9H_2O$) (98%), sodium acetate (CH_3COONa) (99%), sodium hydroxide (NaOH) (98%), formaldehyde (HCHO) (36-38wt% in water), HCl (36-38wt% in water), sodium (anilinomethane) sulfonate (99.9%), aniline (99.9%) and phosphoric acid (85wt. % in water) were supplied by Sinopharm Chemical Reagent Co., Ltd. N,N-Dimethylformamide (DMF) (99.9%), N,N-Dimethylacetamide (DMAc) (99.9%), o-dichlorobenzene (99.9%), tetrahydrofuran (THF) (99.9%), methyl alcohol (99.9%) and ethanol (99.9%) were obtained from Tianjin Fuyu Fine Chemical Co., Ltd.

Powder X-ray diffraction study were done in a Rigaku Ultima IV X-Ray diffractometer using Cu-K α (λ = 0.15406 nm) radiation. Thermal gravimetric analysis (TGA) was used by Mettler Toledo thermal analyzer with a heating rate of 10 ^{o}C min^{-1} in the range of 40-900 ^{o}C under N_2 atmosphere. Infrared (IR) spectra were obtained on a Nicolet 330 FTIR spectrometer. The N_2 adsorption/desorption isotherms and pore size distributions were obtained from surface area analyzer ASAP 2020. The ^{1}H NMR spectrum was collected using a 400 MHz Varian INOVA spectrometer and was referenced to the residual solvent peak. ^{31}P, ^{1}H and ^{13}C spectra solid-state NMR spectra were obtained using a Bruker Avance III model 400 MHz NMR spectrometer at a MAS rate of 5 kHz. Elemental analysis was conducted on a PerkinElmer 240C elemental analyzer for C, H, and N determination. Field-emission scanning electron microscopy

(FESEM) images were obtained on a Hitachi SU-8000 instrument. The energy dispersive X-ray spectroscopy (EDS) mapping was measured on an Oxford INCA X-MAXN 80T. X-ray photoelectron spectroscopy (XPS) spectra were taken from an ESCA250Xi spectrometer. Atomic Force Microscope (AFM) was conducted on the Bruker MultiMode 8 AFM equipped with a NanoScope V controller. The water contact angle tests were carried out on the JC2000 contact angle measuring instrument. The inductively coupled plasma mass (ICP-MS) spectra were recorded using an Optima 8000 spectrometer.

Methods

Synthesis of 4,4'-azodianiline [Azo][1]

p-Nitroaniline (2.76 g, 0.02 mol) was suspended in water (10 ml) at 0-5 ^{o}C. HCl (35%, 4.4 ml, 0.05 mol) and $NaNO_2$ (1.38 g, 0.02 mol) were added slowly, and then the mixture was stirred for 1 h to complete diazotization. In order to prevent the triazine structure, sodium (anilinomethane) sulfonate was used as a coupling component and then hydrolyzed under aqueous alkaline solution to form 4-((4-nitrophenyl)diazenyl)benzenamine.

$NaHSO_3$ (2.08 g, 0.02 mol) was added to the solution of water (40 ml) and ethanol (50 ml), and then heated to 60 ^{o}C. After cooling the reaction mixture to room temperature, aniline (1.86 g, 1.82 ml, 0.02 mol) and HCHO (1.6 ml, 0.02 mol) were added to the solution, followed by stirring for 1 h. Then, the coupling solution was slowly added to the diazotization solution, and stirred at room temperature overnight. After reaction, the pH of the solution was adjusted to 5.0 by adding CH_3COONa, and the crude product was collected by filtration. The intermediate 1 was purified by recrystallization in ethanol (3.6 g, yield 50%). ^{1}H NMR (300 MHz, $CDCl_3$): δ_H = 8.4 (d, 2H), 8.2 (d, 2H), 7.8 (d, 2H), 6.6 (d, 2H), and 5.2 (s, 2H).

The intermediate 1 was dissolved in 1% NaOH aqueous solution, which was then heated under reflux for 4 h. The solution was cooled to room temperature, and 4-((4-nitrophenyl) diazenyl) benzenamine was collected by filtration. The terminal nitro

group was reduced using equivalent sodium sulfide nonahydrate to have amino end groups at both ends. 4-((4-Nitrophenyl) diazenyl) benzenamine (0.23 g, 1 mmol) was dissolved in ethanol (20 ml). $Na_2S\ 9H_2O$ (0.48 g, 2 mmol) in water (20 ml) was added dropwise, and then the combined solution was refluxed for 3 h. On completion of the reaction, the mixture was poured into cold water (100 ml). Precipitate 4,4′-azodianiline (2) was filtered off, washed with water, and dried in vacuo (0.15 g, yield 65%). ^1H NMR (300 MHz, DMSO): δ_H = 7.6 (d, 2H), 6.7 (d, 2H), 5.7 (s, 4H).

Synthesis of CD-Azo

α-Cyclodextrin [α-CD] (3.0 g, 3.1 mmol) and 4,4'-azodianiline [Azo] (100 mg, 0.47 mmol) were added to an aqueous sodium hydroxide solution (25 mL) at pH=10, and the mixture was stirred for 4 hours at room temperature. Collect the powder by filtration and continue washed with a large amount of water, which was isolated by filtration and dried at 80 ^oC under vacuum for 12 hours to afford the tan powder of CD-Azo in ~81 % yield. Element Anal. Calcld. For $C_{24}H_{36}N_2O_{15}$: C, 48.65; H, 6.12; N, 4.73; found: C, 48.28; H, 6.21; N, 4.49.

Synthesis of TpAzo[2]

In the typical synthesis, a Pyrex tube (o.d. i.d. = 10×8 mm^2 and length 18 cm) is charged with 1,3,5-triformylphloroglucinol [Tp] (63 mg, 0.3 mmol), 4,4′-azodianiline [Azo] (95.5 mg, 0.45 mmol), 3 mL of DMA and 3 mL of o-dichlorobenzene. This mixture was sonicated for 10 minutes in order to get a homogeneous dispersion. The tube was then flash frozen at 77 K (liquid N_2 bath) and degassed by three freezepump-thaw cycles. The tube was sealed off and then heated at 120 ^oC for 3 days. A dark red coloured precipitate was collected by filtration and washed with DMA and water thrice, which was isolated by filtration and washed with anhydrous THF and anhydrous methyl alcohol for 24 hours respectively. Then dried at 100 ^oC under vacuum for 12 hours to get corresponding COFs in ~80 % isolated yield. Element Anal. Calcld. For $C_9H_6N_2O$: C, 68.35; H, 3.82; N, 17.71; found: C, 67.28; H, 3.91; N, 17.29.

Synthesis of CD-TpAzo

In the typical synthesis, a 10 mL glass vial is charged with 1,3,5-triformylphloroglucinol [Tp] (21mg, 0.1 mmol), CD-Azo (177.8mg, 0.15 mmol), 3 mL of DMA and 3 mL of o-dichlorobenzene. This mixture was sonicated for 10 minutes in order to get a homogeneous dispersion. The vial was sealed off and then heated at 100 ^{o}C for 10 hours. A dark red coloured precipitate was collected by filtration and washed with DMA and water thrice, which was isolated by filtration and washed with anhydrous THF and anhydrous methyl alcohol for 24 hours respectively. Then dried at 100 ^{o}C under vacuum for 12 hours to get corresponding COFs in ~75 % isolated yield. Element Anal. Calcld. For $C_{27}H_{36}N_2O_{16}$: C, 50.31; H, 5.63; N, 4.35; found: C, 50.11; H, 5.66; N, 4.92.

Synthesis of TpAzo@H_3PO_4-x and CD-TpAzo@H_3PO_4-x

A series of experiments were conducted to explore the H_3PO_4 doped maximum by grinding different amounts (5, 10, and 13 μL) of H_3PO_4 into TpAzo (10 mg). TpAzo@H_3PO_4-13 was obtained by adding 85% phosphoric acid (13 μL) into TpAzo (10 mg), grinding for half an hour, and then drying under vacuum at 120 ^{o}C for 12 hours.

The same procedure is executed to explore the H_3PO_4 doping maximum by grinding different amounts (5, 10, 15 and 18 μL) of H_3PO_4 into CD-TpAzo (10 mg). CD-TpAzo@H_3PO_4-18 was obtained by adding 85% phosphoric acid (18 μL) into CD-TpAzo (10 mg), grinding for half an hour, and then drying under vacuum at 120 ^{o}C for 12 hours.

Section S-2: Experimental Procedures

Proton conductivity measurement

The as-synthesized sample was placed in mold and pressed into a pellet with a diameter of 3 mm and a thickness range of 1-2 mm by a tableting machine. The pellet was placed in the center of the glass pellet and fixed horizontally with two 20 cm of gold wires, and two sides of the pellet were coated with silver glue, and then waited for about 30 minutes to dry. Impedance analysis was performed with a 1260A Impedance/Gain-Phase Analyzer from 10 MHz to 0.1 Hz with an input voltage 200 mV in a constant temperature and humidity, which were controlled using a BPS-50CL humidity control chamber. Each sample was pressed at least three tablets, and repeated cyclic tests were performed on each tablet. Typically, the impedance at each temperature were measured after equilibration for a period of 6-10 hours. The resistance values were obtained by fitting the impedance profile using zview software. The circuit equivalent used for fitting is as follows:

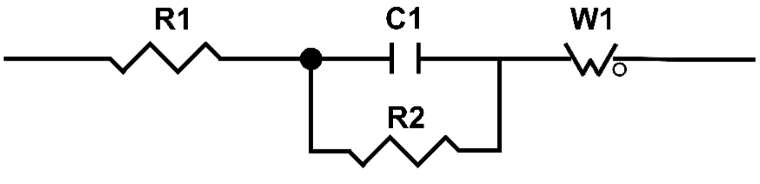

R1 corresponds to the resistances of wire and electrode, while R2 accounts for the bulk resistance of the pellet. Proton conductivity (σ, S cm^{-1}) of each sample was obtained by the following equation:

$$\sigma = \frac{l}{RS}$$

Where l and S are the length (cm) and area (cm^2) of the pellet, respectively, and R is the intrinsic resistance value (Ω) of the material fitted by the equivalent circuit of the first semicircle using zview software. The activation energy (E_a) of the material is estimated according to the following Arrhenius equation:

$$\sigma T = \sigma_0 \, exp(-\frac{E_a}{k_B T})$$

Where σ_0 is the pre-exponential factor, T is the temperature, and k_B is the Boltzmann constant.

Spin-lattice relaxation test

Spin lattice relaxation were tested using a Bruker Avance III model 400 MHz NMR spectrometer at a MAS rate of 5 kHz. The relaxation process, which arises due to the interaction between matter, is the process by which the macroscopic magnetization vector of a spin-nucleon population system is out of equilibrium after receiving an Radio Frequency (RF) field excitation, and when the RF stops, the nucleon population returns from the nonequilibrium state to the equilibrium state. The relaxation process includes longitudinal relaxation (also called spin-lattice relaxation) and transverse relaxation (also called spin-spin relaxation). The relaxation times corresponding to these two relaxation processes are denoted by T_1 and T_2, respectively. The one we are testing is the longitudinal relaxation. The essence of the longitudinal relaxation process is the return to the ground state of the RF energy absorbed during the release of excitation from the spin nucleus, marked by the recovery of M_z, and the speed of the process measured by the time taken for its recovery. Theoretically, according to Bloch's equation, the curve of M_z with time follows a single-exponential decay law:

$$M_z = M_0\left[1 - e^{-t/T_1}\right]$$

M_z is the longitudinal magnetization vector at moment t of the start of the spin-lattice relaxation, M_0 is the maximum longitudinal magnetization vector, and T_1 is the spin-lattice relaxation time. The time required for the longitudinal magnetization vector to recover from zero to 67% (1-1/e) of the maximum value is defined as T_1.

Preparation of COF-Nafion membrane[3]

Hybrid films were prepared by solution casting. The first step is to heat the commercial 20% Nafion PFSA DuPont D2020 dispersion at 80 ℃ for 2 h, evaporate the solvent to obtain dry Nafion resin, and continuously stir in DMF for 1 h to obtain DMF treated Nafion. Then, the weighed CD-TpAzo@H_3PO_4-18 was dispersed in DMF, stirred for 6 h, and ultrasonically stirred for 1 h. Finally, the Nafion treated with DMF and the DMF solution containing COF were stirred vigorously for 2 h to obtain the casting solution, which was poured into a glass Petri dish with an internal diameter of 9 cm and stored at 80 ℃ for 10 h. The obtained membrane was designated as 5% CD-TpAzo@H_3PO_4-18/Nafion, where 5% is the weight percentage of CD-TpAzo@H_3PO_4-18 and Nafion matrix.

Preparation of fuel cell components

5% CD-TpAzo@H_3PO_4-18@Nafion was selected to prepare the membrane electrode assembly. Commercial Pt/C catalyst (40 wt.%, Johnson Matthey) was sprayed onto a carbon cloth gas diffusion layer (ELAT GDL-LT 1200W) and used as electrode. The catalyst was dispersed in a mixture of deionized water and isopropyl alcohol (3 : 7 v/v), and then treated with ultrasound at 0 ^{o}C for 3 h in a water bath. The catalyst was then brushed onto the gas diffusion layer to obtain the electrode. The catalyst was Pt/C with 0.5 mg cm^{-2} for the anode and 0.5 mg cm^{-2} for the cathode. The mixed membrane is sandwiched between the two gas-diffusion electrodes to obtain a single cell with an effective area of 2.5 ×2.5 cm^2, and hot-pressed into a membrane electrode assembly

(MEA) at 4 MPa and 120 ^{o}C for 5 min. The fuel cell test station (Dilian Yuke, YK-A10) operates at 80 ^{o}C and 100% relative humidity (RH). Humidified H_2 and O_2 were injected into the anode and cathode, respectively, at a flow rate of 50 mL min^{-1}. Back pressures of 1 bar were applied to both anode and cathode.

Computational details

Stacking energies are thus calculated as E(bilayer) – [E(top) + E(bottom)]. Energy minimized crystal cell bilayer (top and side views) of TpAzo and CD-TpAzo based on Firstprinciples calculations performed within the framework of density functional theory (DFT). Energies of each layer and the bilayer were calculated in the Vienna ab initio simulation package (VASP) code, which is based on the projector-augmented wave (PAW) method. To describe the exchange correlation interactions, a generalized gradient approximation (GGA) with the Perdew-Burke-Ernzerhof (PBE) functional was employed. Along the z-direction, a large vacuum space of 20 Å was added to avoid interactions between the nearest-neighbor unit cells. The plane-wave cutoff energy was set to 400 eV. For geometry optimizations, the Brillouin zone was sampled with the Gamma centered Monkhorst-Pack scheme K-point grid of 1 × 1 × 1. All the structures were optimized using the conjugant gradient method with an energy convergence criterion of 1×10^{-5} eV and a force convergence criterion of 0.02 eV/Å. A long-range dispersion corrected DFT-D3 method proposed by Grimme was incorporated to describe the van der Waals interactions.

Gas permeation measurements

Single (H_2) gas permeation tests were performed with the Wicke-Kallenbach permeation technique. In order to reduce the influence of reverse diffusion on the feed side, argon was used as the sweep gas. The trans-membrane partial pressure difference of component i (ΔP_i) was set from 1.4 to 1.8 bar (1.4-1.8 bar for feed gas i upstream and 1.0 bar for the sweep gas of Ar downstream). The sweep gas was set to a flow rate of 40 mL min^{-1} to minimize concentration polarization on the permeate side, and the flow rate of the feed

gas was set at 100 mL min^{-1}. The permeate gas composition was analyzed using a gas chromatograph (SHIMADZU GC-2014C). The working curve was obtained by fitting 6-8 points, and each point was based on the results of more than 20 parallel tests. The gas permeance (Barrer, 1 Barrer = 3.35 × 10^{-16} mol m^{-1} s^{-1} Pa^{-1}) was calculated using the following equation

$$\text{Permeance}_i = N_i/(\Delta P_i \times A)$$

in which N_i (mol s^{-1}) is the permeate flow rate of component i, ΔP_i (Pa) is the trans-membrane pressure drop of i, and A (m^2) is the membrane area.

Molecular dynamics simulation

The molecular dynamics simulations were performed using the Materials Studio software. A COF model was constructed with dimensions of a = b = 31.5 Å, c = 33 Å, α = β = 90°, γ = 120°, incorporating H_3PO_4, $H_2PO_4^-$and H^+. A CD-COF model was also built with dimensions of a = b = 31.5 Å, c = 32 Å, α = β = 90°, γ = 120°, containing H_3PO_4, $H_2PO_4^-$ and H^+, to maintain electrical neutrality within the system. Among them, the dissociation degree of H_3PO_4 is set at 10%.

First, geometric optimization was conducted using the Smart algorithm. Subsequently, molecular dynamics simulations were carried out in the NVT ensemble at a temperature of 150 ℃, with temperature control managed by the NHL method. The time step was set to 0.5 fs, and the total simulation time was 1 ns. Trajectory frames were output every 1 ps for result analysis.

The COMPASS force field was employed throughout the calculations. The Ewald summation method was used to describe electrostatic interactions, while van der Waals interactions were treated using the Atom-based method, with a cutoff radius set to 12.5 Å. For hydrogen bond analysis, the maximum hydrogen-acceptor distance was set to 2.5 Å, and the minimum donor-hydrogen-acceptor angle was defined as 120 °.

Section S-3: Preparation of precursors (CD-Azo)

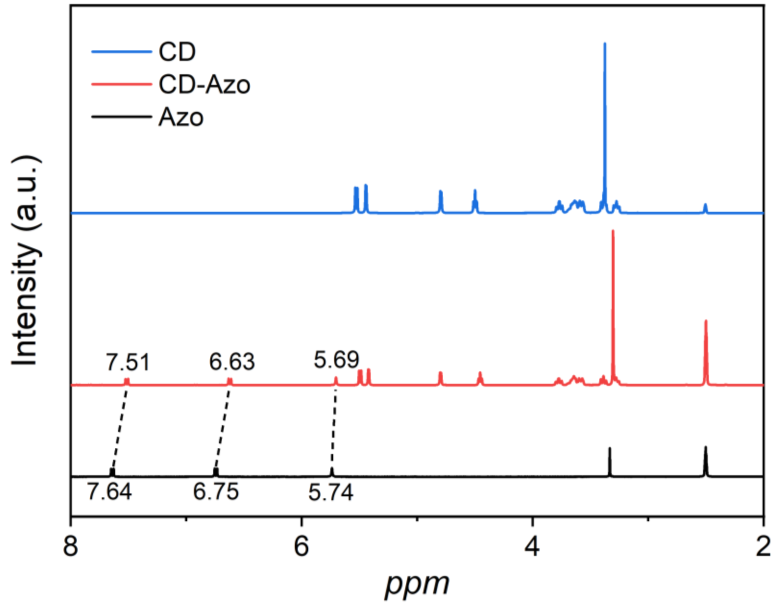

Figure S1. ^{1}H NMR spectra of 4,4'-azodianiline (Azo), α-cyclodextrin (CD) and CD-Azo in DMSO at 500 MHz.

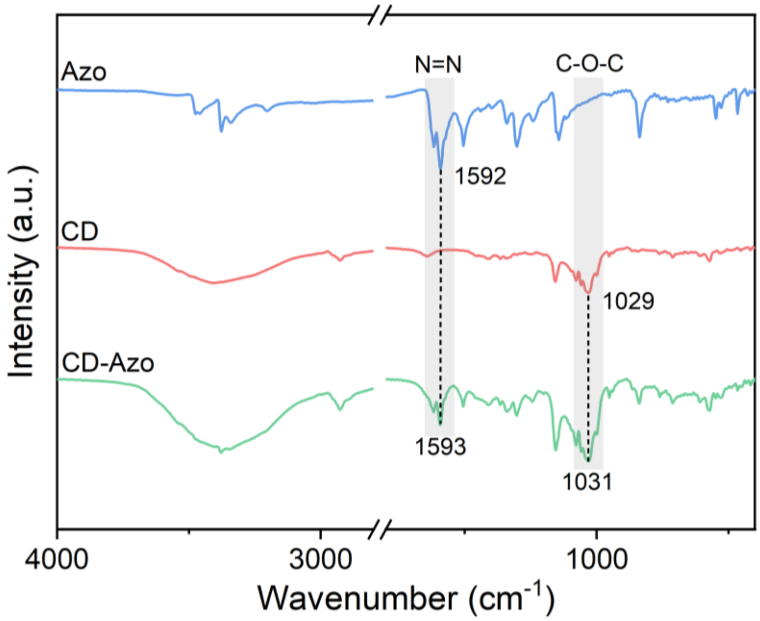

Figure S2. FT-IR spectra of Azo, CD, and CD-Azo.

Section S-4: Characterization of the as-synthesized COFs

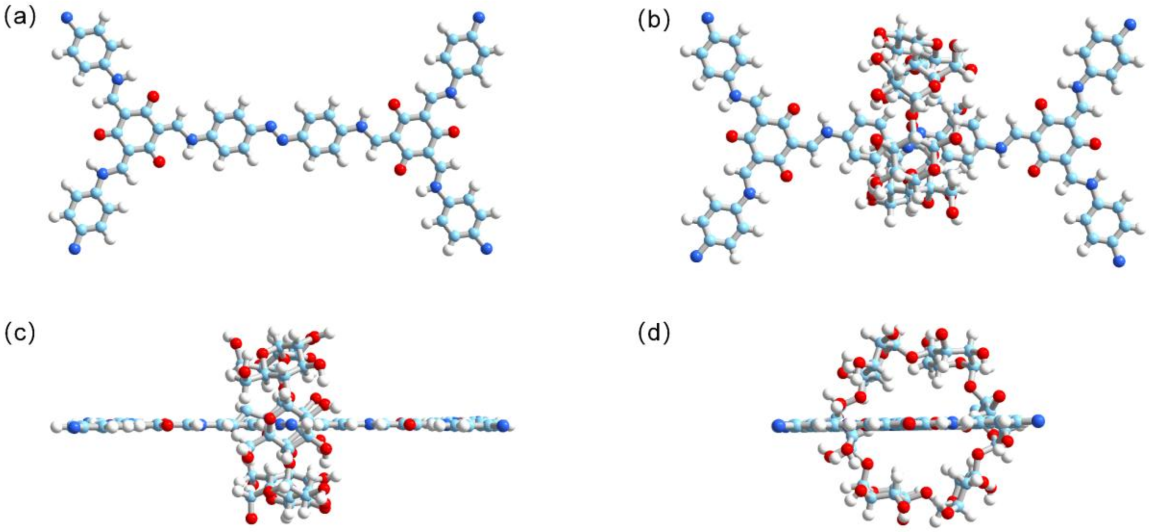

Figure S3. The structural units of TpAzo (a) and CD-TpAzo (b), side view of CD-TpAzo (c) and (d).

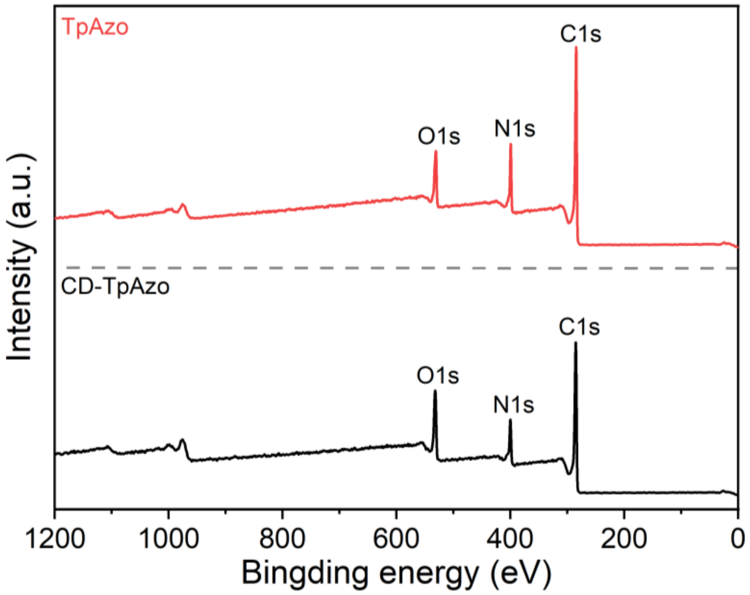

Figure S4. XPS profiles of CD-TpAzo (bottom) and TpAzo (top).

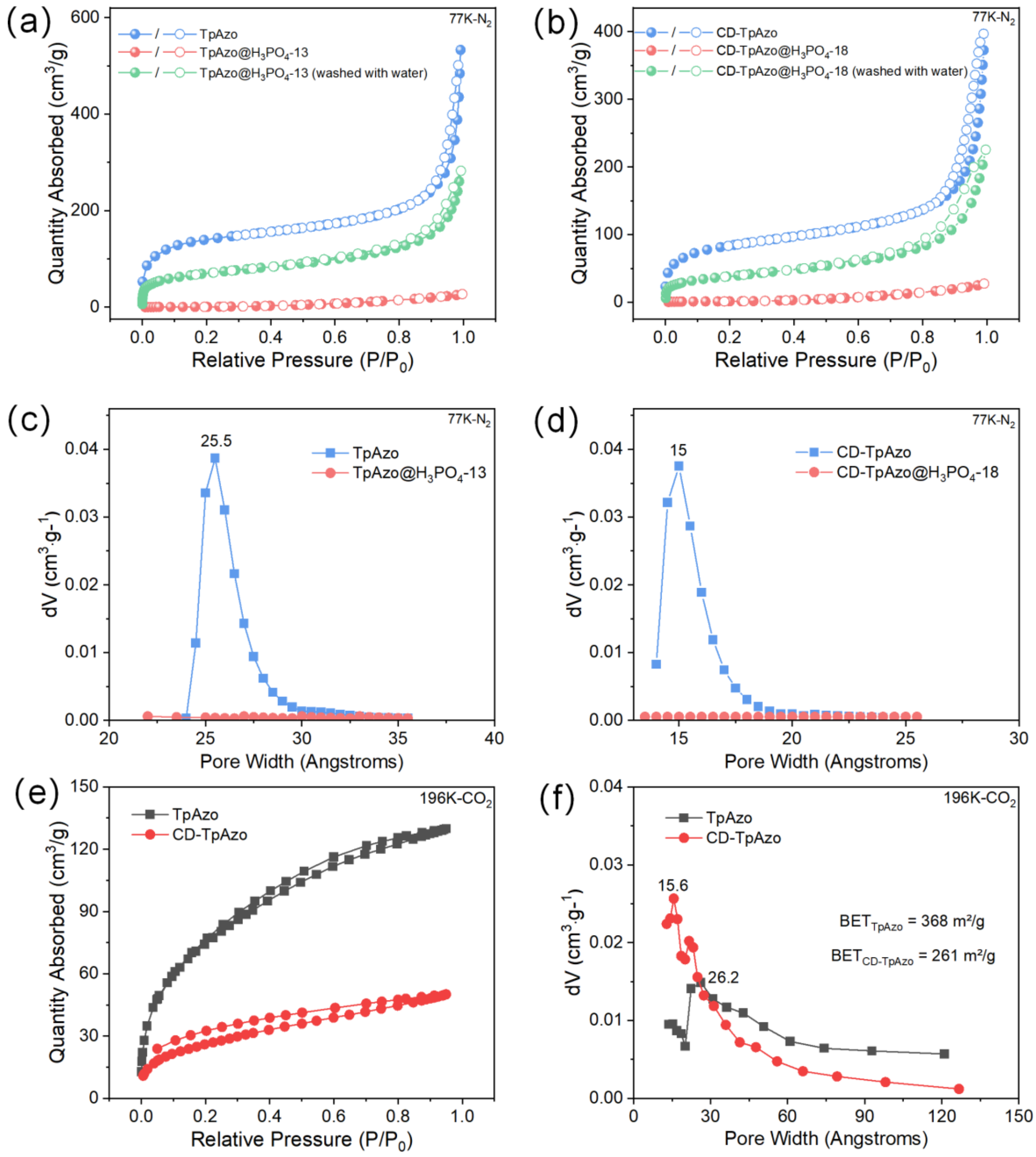

Figure S5. Nitrogen sorption isotherm profiles of (a) TpAzo, TpAzo@H_3PO_4-13 and TpAzo@H_3PO_4-13 after washing with water, (b) CD-TpAzo, CD-TpAzo@H_3PO_4-18 and CD-TpAzo@H_3PO_4-18 after washing with water, pore size distribution of Tp-Azo (c), CD-TpAzo (d). CO_2 sorption isotherm profiles (e), pore size distribution (f) of TpAzo and CD-TpAzo at 196 K.

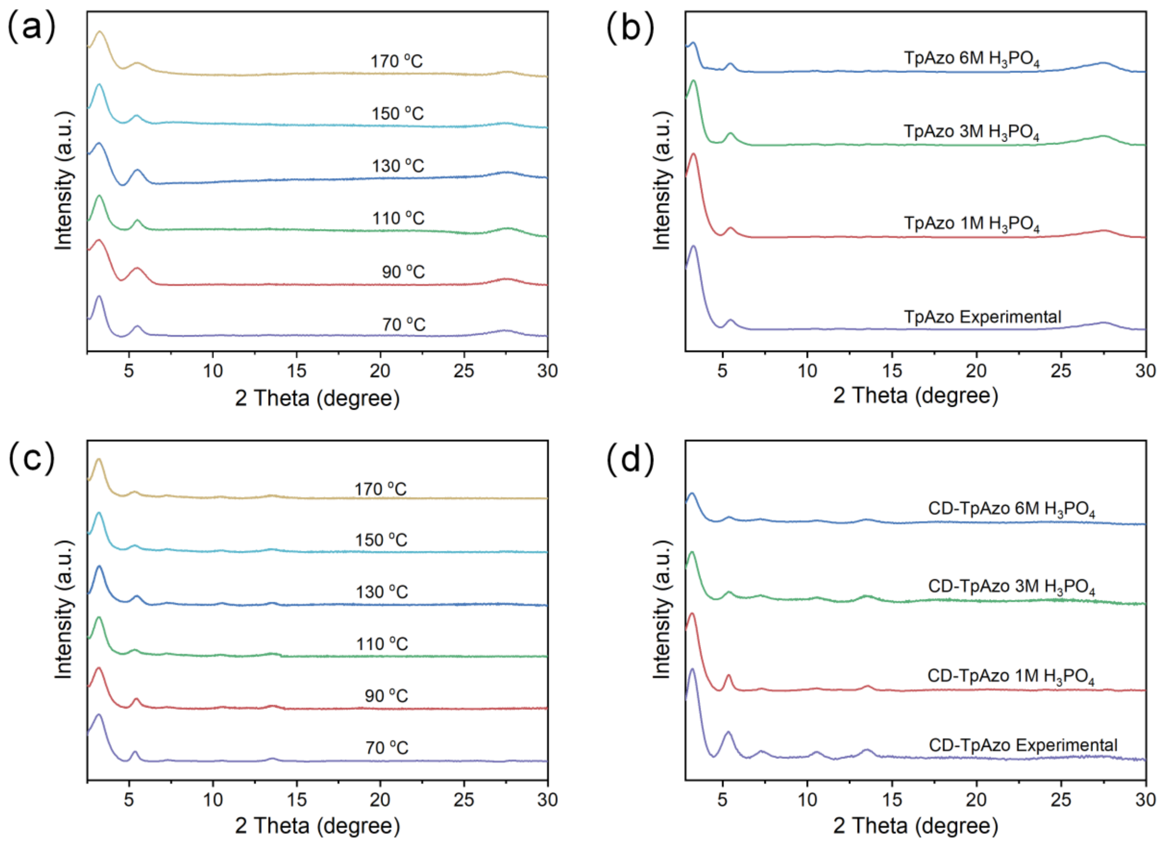

Figure S6. PXRD patterns of TpAzo (a) after treating 24 hours at different temperatures; (b) after treating 3 days in different concentrations of acids; PXRD patterns of CD-TpAzo (c) after treating 24 hours at different temperatures; (d) after treating 3 days in different concentrations of acids.

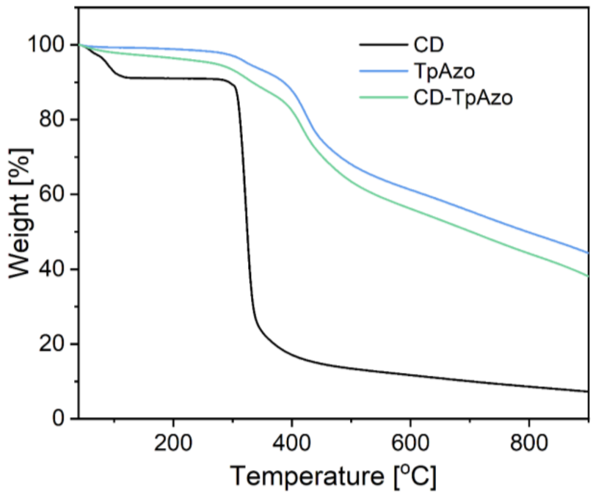

Figure S7. Thermogravimetric (TGA) spectra of CD, TpAzo and CD-TpAzo.

Section S-5: The characterization of the phosphoric acid doped COF samples

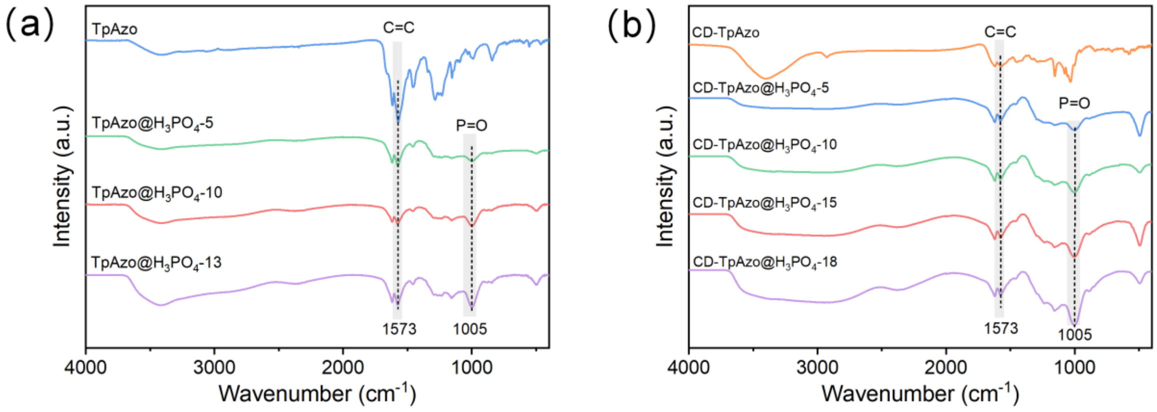

Figure S8. FT-IR spectra of (a) TpAzo, TpAzo@H_3PO_4-5, TpAzo@H_3PO_4-10 and TpAzo@H_3PO_4-13, (b) CD-TpAzo, CD-TpAzo@H_3PO_4-5, CD-TpAzo@H_3PO_4-10, CD-TpAzo@H_3PO_4-15 and CD-TpAzo@H_3PO_4-18.

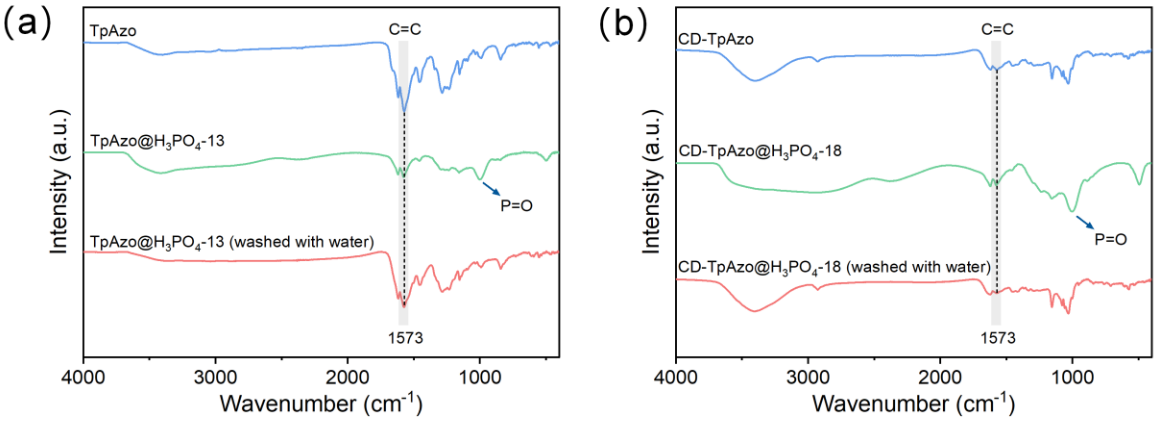

Figure S9. FT-IR spectra of (a) TpAzo, TpAzo@H_3PO_4-13 and TpAzo@H_3PO_4-13 (washed with water), (b) CD-TpAzo, CD-TpAzo@H_3PO_4-18 and CD-TpAzo@H_3PO_4-18 (washed with water).

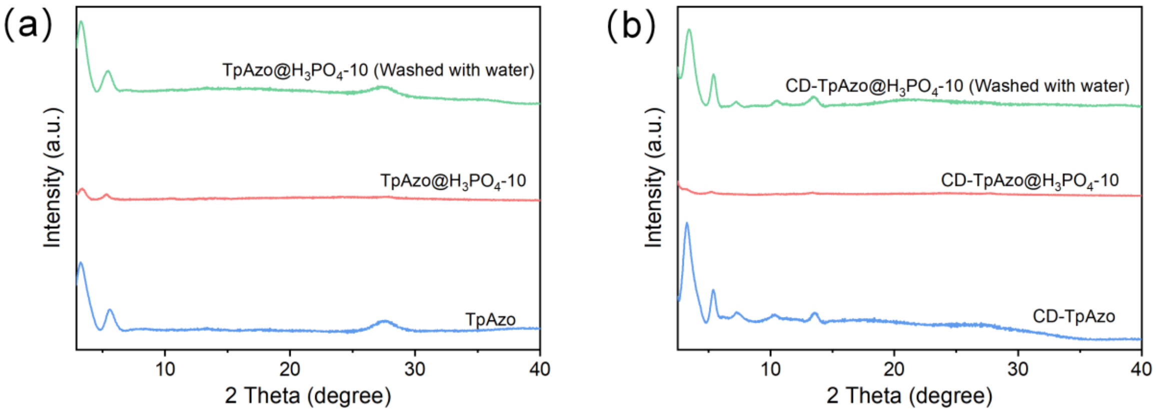

Figure S10. The PXRD patterns of (a) TpAzo, TpAzo@H_3PO_4-13 and TpAzo@H_3PO_4-13 (washed with water), (b) CD-TpAzo, CD-TpAzo@H_3PO_4-18 and CD-TpAzo@H_3PO_4-18 (washed with water).

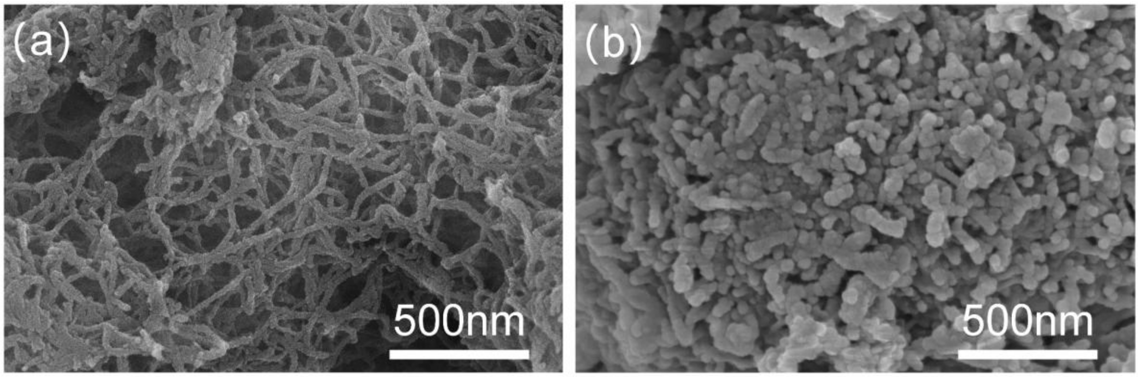

Figure S11. SEM images of (a) TpAzo and (b) CD-TpAzo.

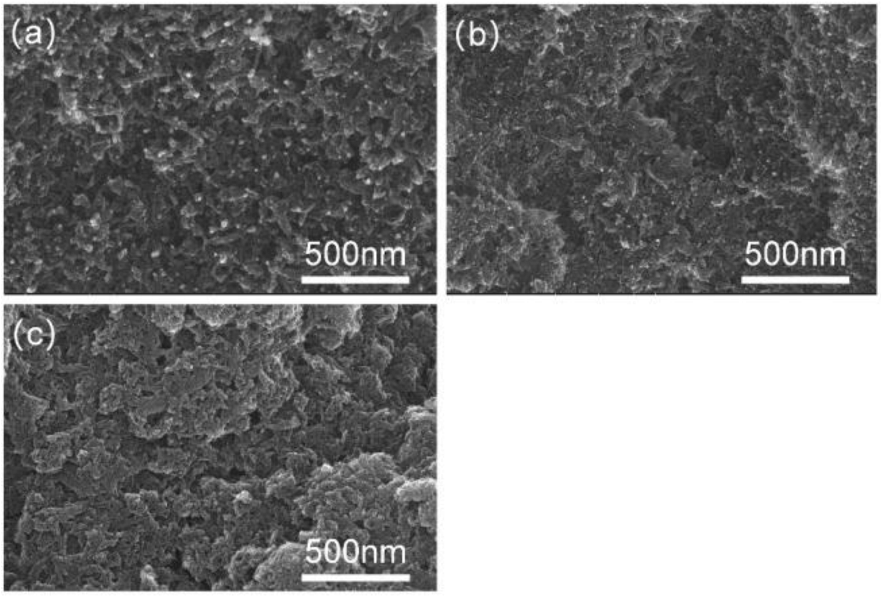

Figure S12. SEM images of (a) TpAzo@H_3PO_4-5, (b) TpAzo@H_3PO_4-10 and (c) TpAzo@H_3PO_4-13.

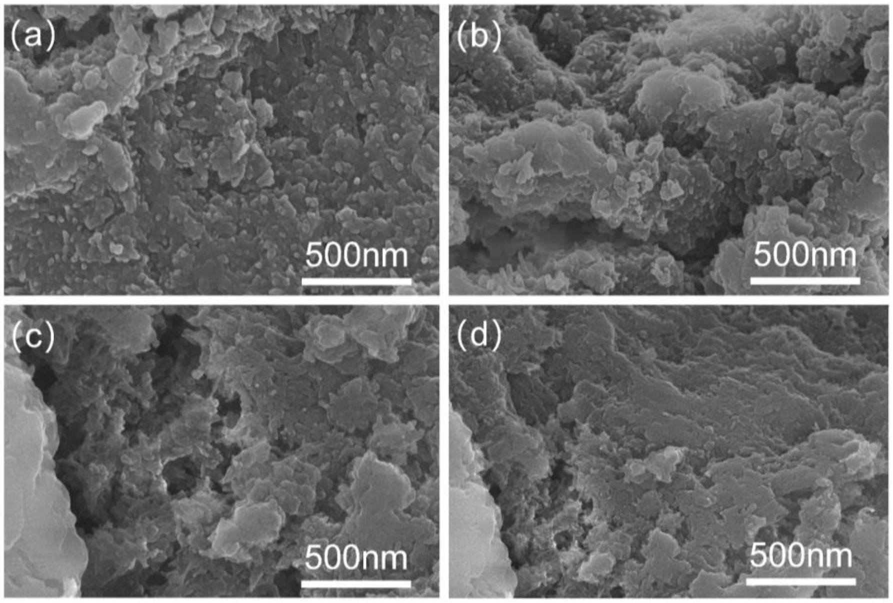

Figure S13. SEM images of (a) CD-TpAzo@H_3PO_4-5, (b) CD-TpAzo@H_3PO_4-10, (c) CD-TpAzo@H_3PO_4-15, and (d) CD-TpAzo@H_3PO_4-18.

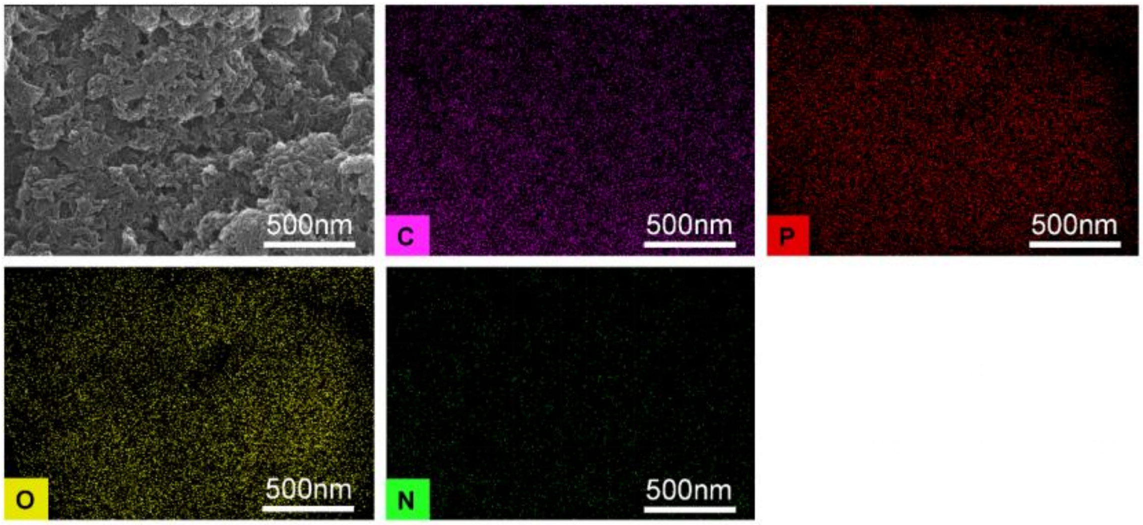

Figure S14. Scanning electron microscopy (SEM) image and the corresponding mapping images of TpAzo@H_3PO_4-13.

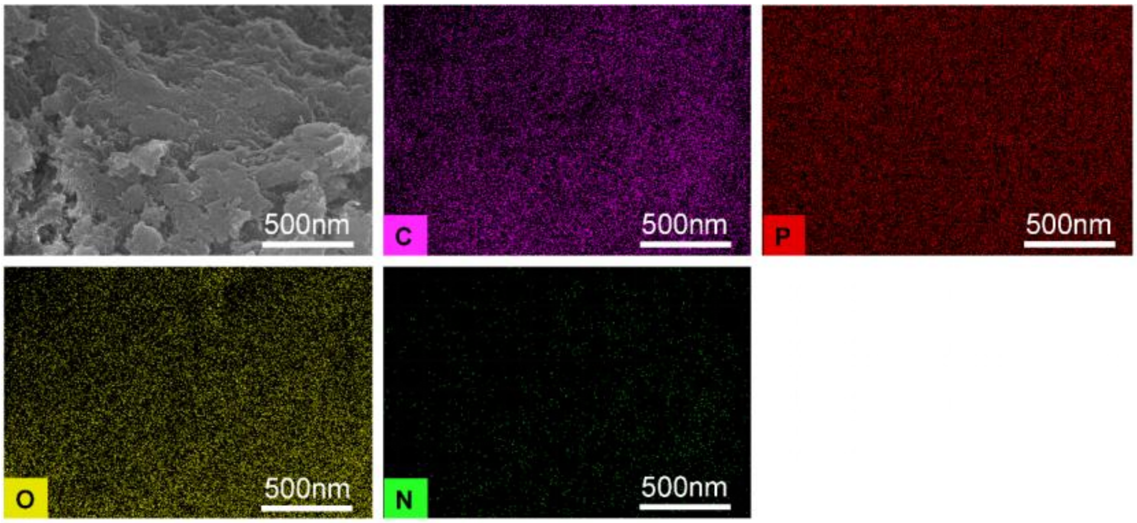

Figure S15. Scanning electron microscopy (SEM) image and the corresponding mapping images of CD-TpAzo@H_3PO_4-18.

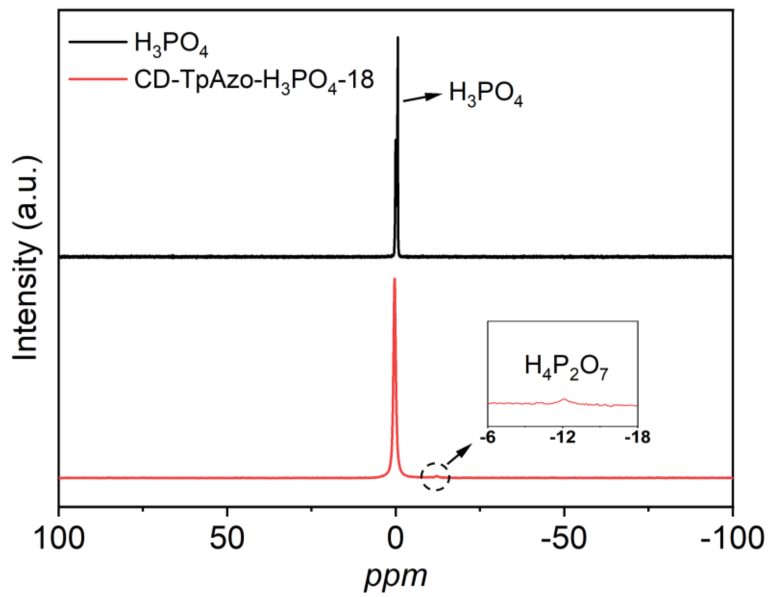

Figure S16. Liquid-state ^{31}P NMR spectrum of H_3PO_4 and solid-state ^{31}P NMR spectrum of CD-TpAzo@H_3PO_4-18.

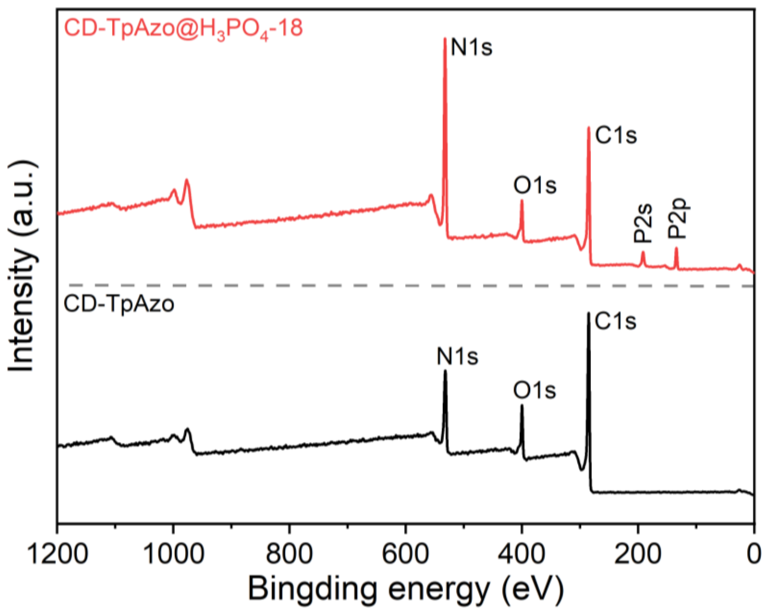

Figure S17. XPS profiles of CD-TpAzo (bottom) and CD-TpAzo@H_3PO_4-18 (top).

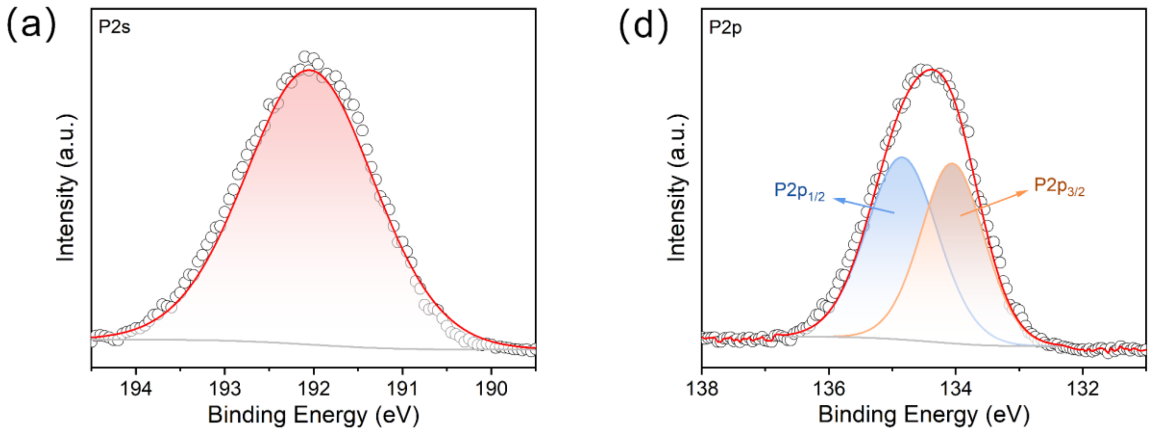

Figure S18. The high-resolution XPS spectra of P 2s (a) and P 2p (b) for CD-TpAzo@H_3PO_4-18.

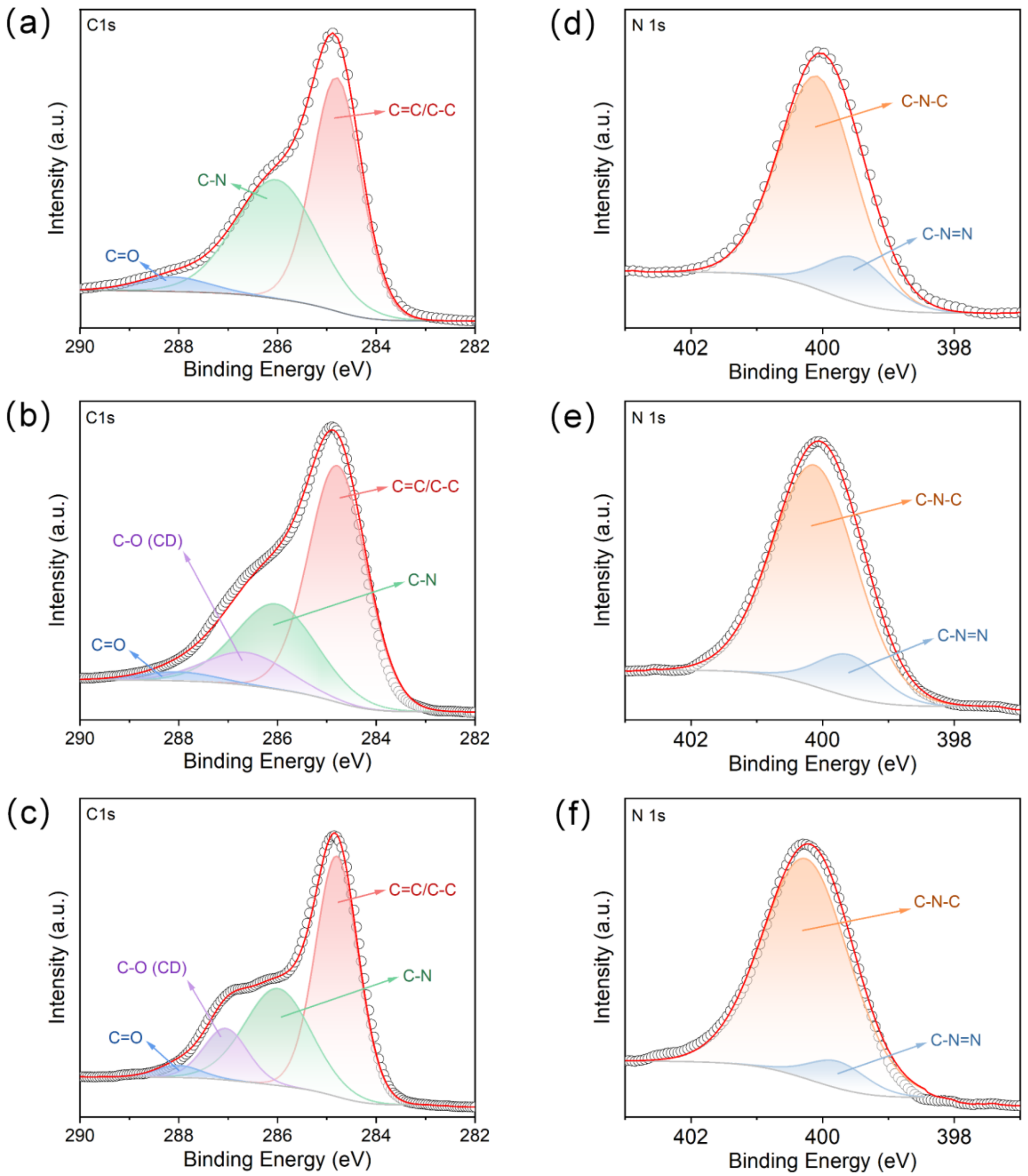

Figure S19. The high-resolution XPS spectra of C 1s for (a) TpAzo, (b) CD-TpAzo and (c) TpAzo@H_3PO_4-13, the high-resolution XPS spectra of N 1s for (d) TpAzo, (e) CD-TpAzo and (f) CD-TpAzo@H_3PO_4-18.

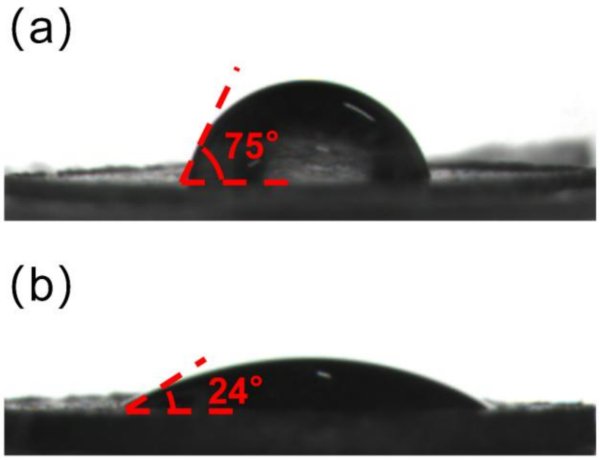

Figure S20. The water contact angles of TpAzo (a) and CD-TpAzo (b).

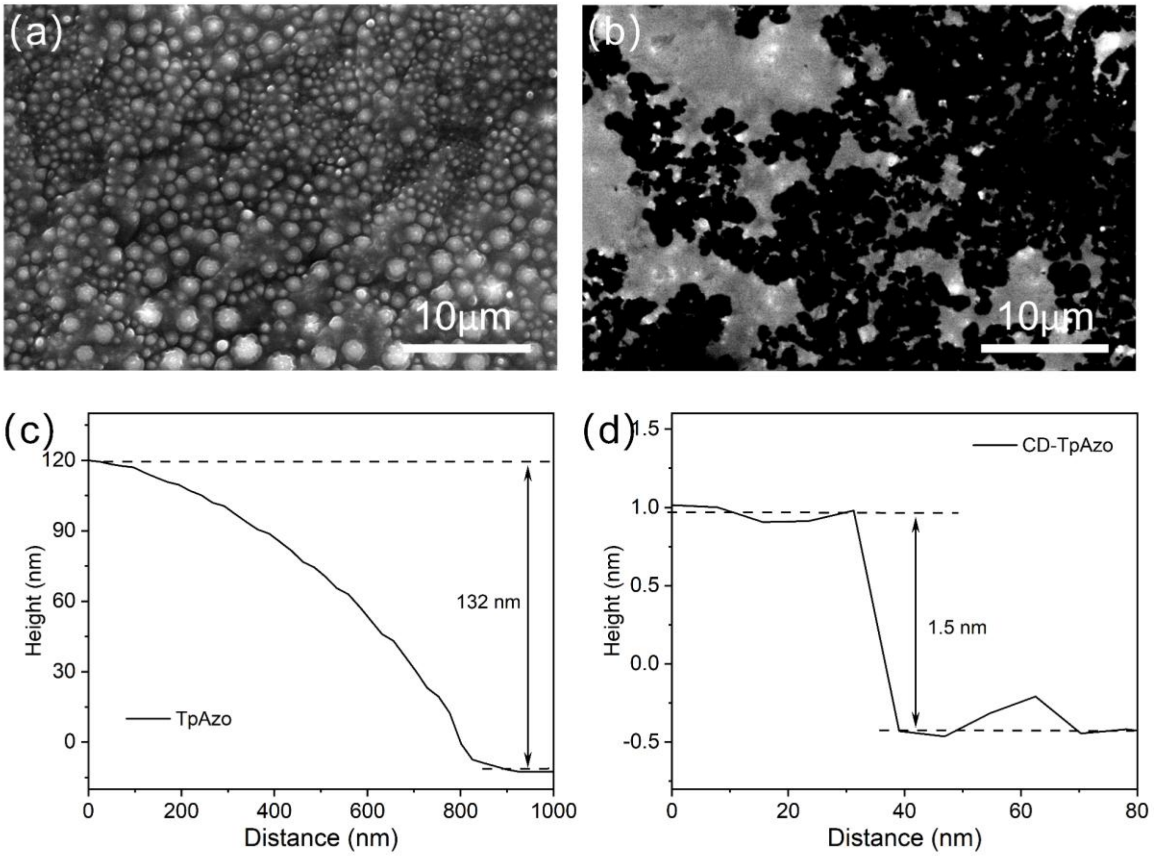

Figure S21. The SEM images of TpAzo (a) and CD-TpAzo (b), and the AFM scanning corresponding section height examples of (c) TpAzo and (d) CD-TpAzo after ultrasonic treatment for 30 minutes in 5% dilute H_3PO_4 solution.

Section S-6: The measurements of anhydrous proton conductivity

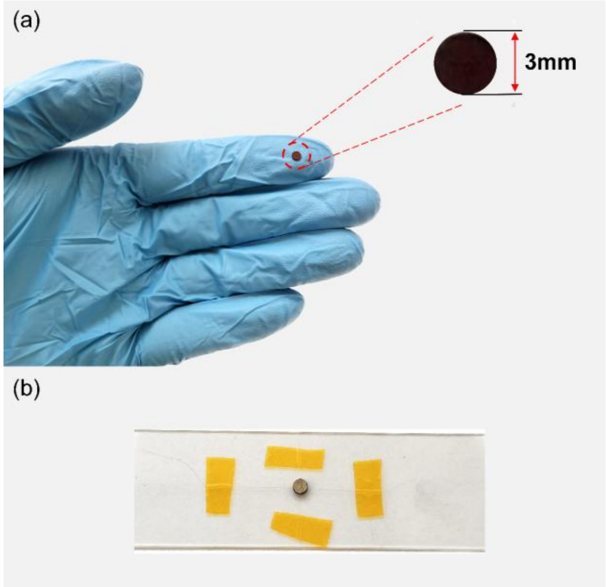

Figure S22. (a) The digital image of the pellet of CD-TpAzo@H_3PO_4-18 used for proton conductivity measurements, (b) the pellet of CD-TpAzo@H_3PO_4-18 with conductive adhesive and gold wire used for proton conductivity measurements.

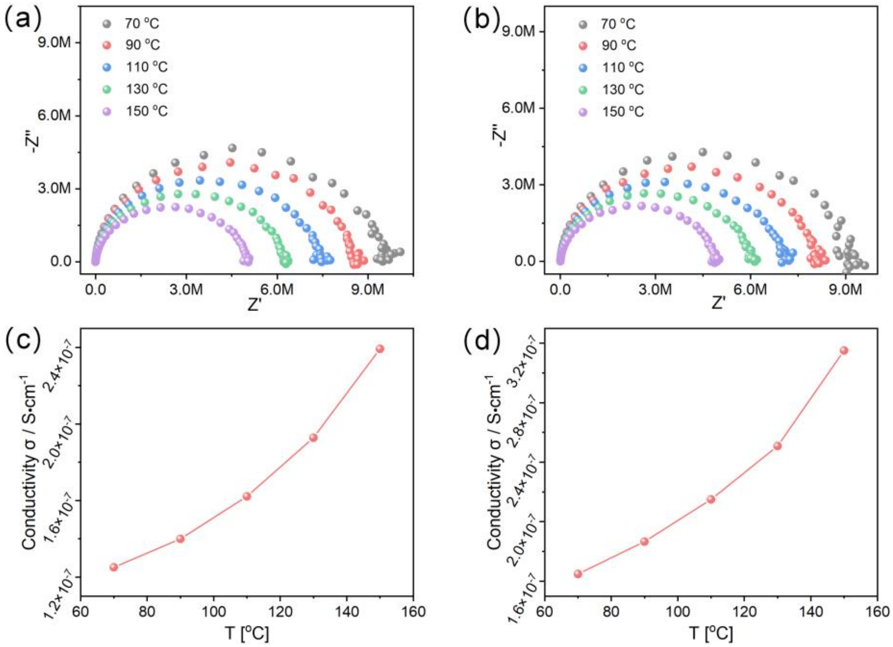

Figure S23. Nyquist plot of TpAzo (a) and CD-TpAzo (b) under various temperatures, temperature-dependent proton conductivity for TpAzo (c) and CD-TpAzo (d).

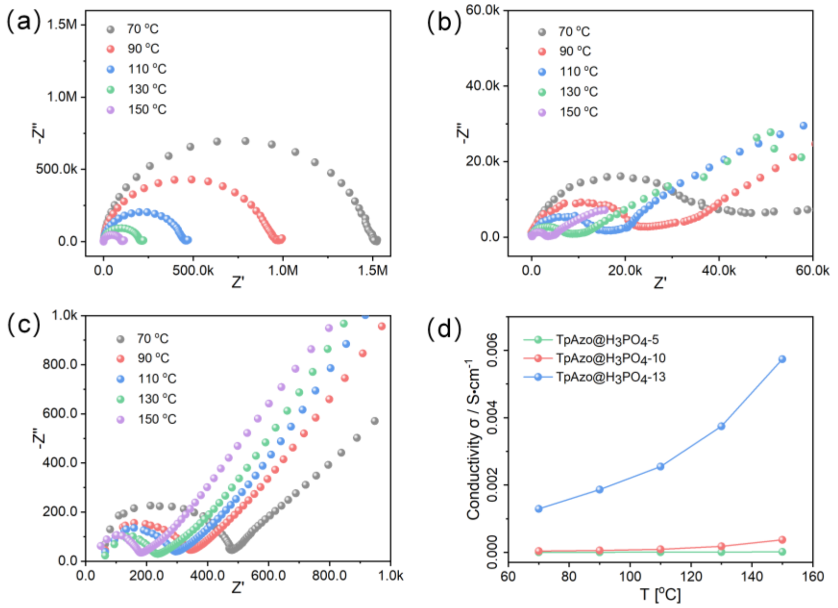

Figure S24. Nyquist plot of TpAzo@H_3PO_4-5 (a), TpAzo@H_3PO_4-10 (b) and TpAzo@H_3PO_4-13 (c) under various temperatures, (d) temperature-dependent proton conductivity for TpAzo@H_3PO_4-5, TpAzo@H_3PO_4-10 and TpAzo@H_3PO_4-13.

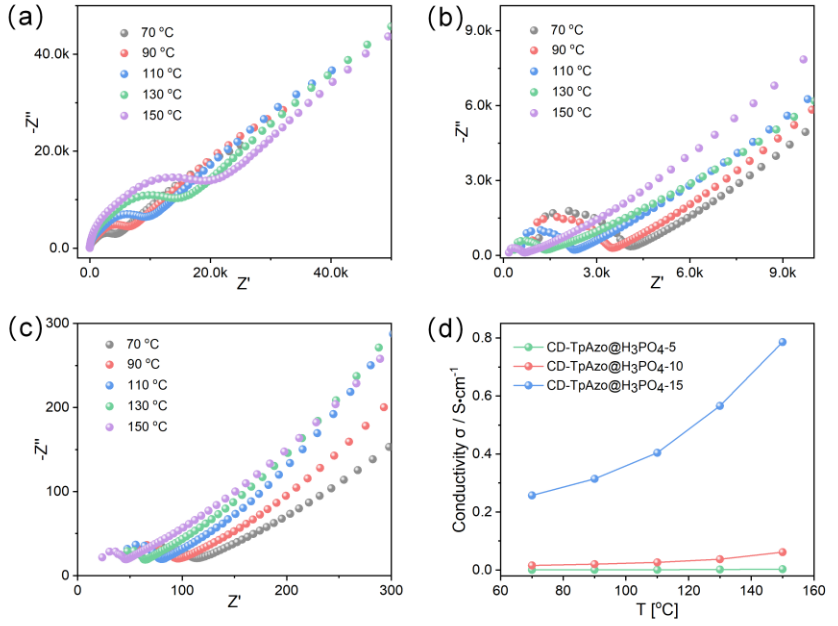

Figure S25. Nyquist plots of CD-TpAzo@H_3PO_4-5 (a), CD-TpAzo@H_3PO_4-10 (b) and CD-TpAzo@H_3PO_4-15 (c) under various temperatures, (d) temperature-dependent proton conductivity for CD-TpAzo@H_3PO_4-5, CD-TpAzo@H_3PO_4-10 and CD-TpAzo@H_3PO_4-15.

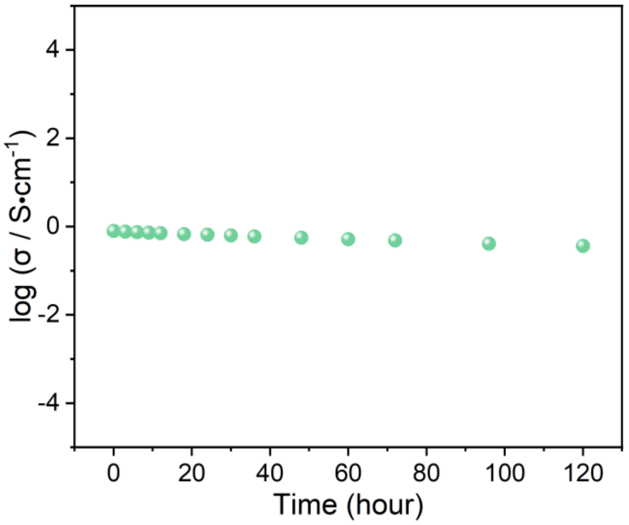

Figure S26. Long-period measurement of CD-TpAzo@H_3PO_4-18 at 150 ℃.

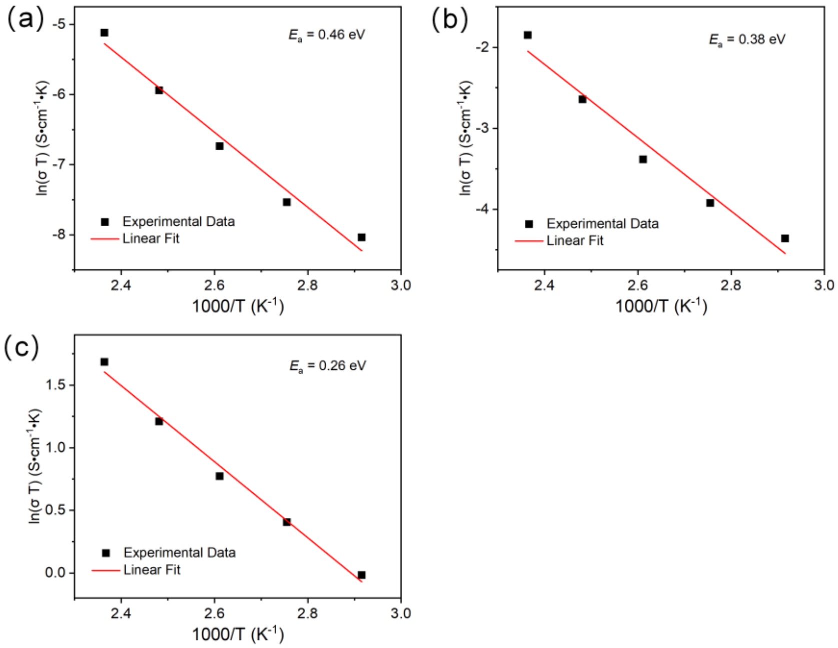

Figure S27. Arrhenius plots of TpAzo@H_3PO_4-5 (a), TpAzo@H_3PO_4-10 (b) and TpAzo@H_3PO_4-13 (c) under different temperature.

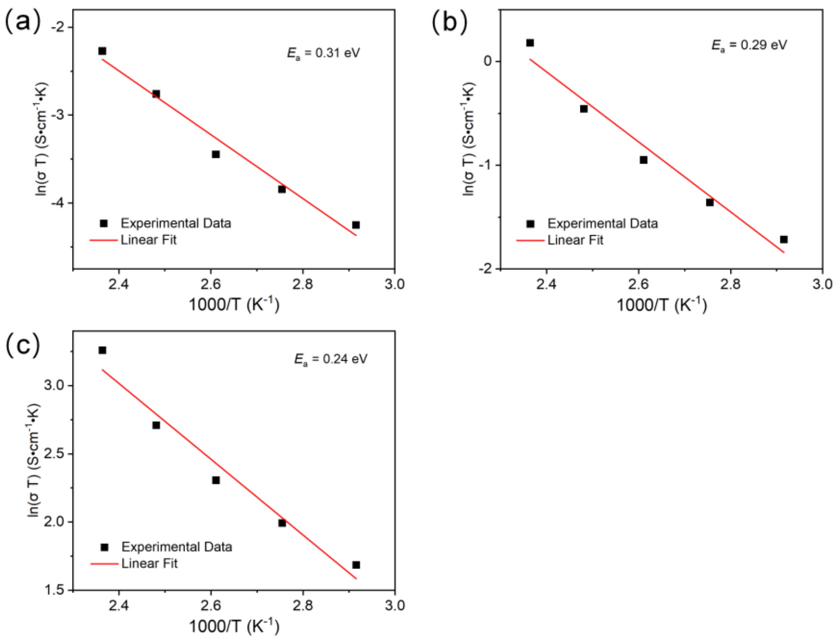

Figure S28. Arrhenius plots of CD-TpAzo-5 (a), CD-TpAzo@H_3PO_4-10 (b) and CD-TpAzo@H_3PO_4-15 (c) under different temperature.

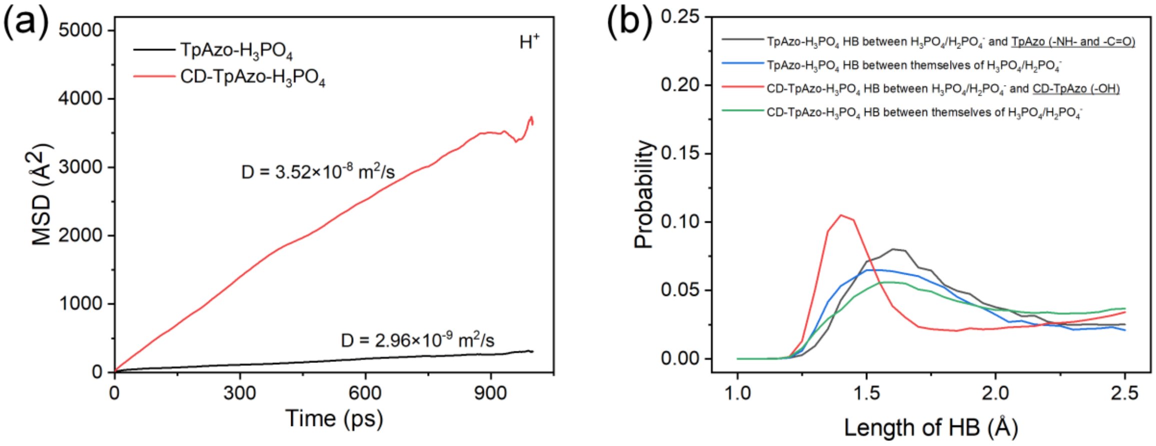

Figure S29. (a) Mean square displacement (MSD) and diffusion coefficients for H^+ in TpAzo@H_3PO_4 and CD-TpAzo@H_3PO_4, (b) distribution probabilities of Hydrogen Bonds (HB) of different lengths in the model.

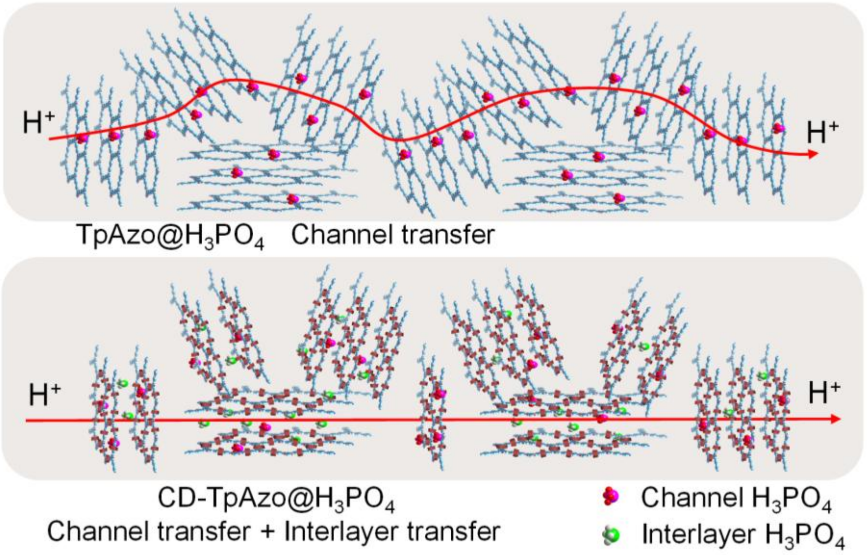

Figure S30. The schematic diagram of proton transfer pathways.

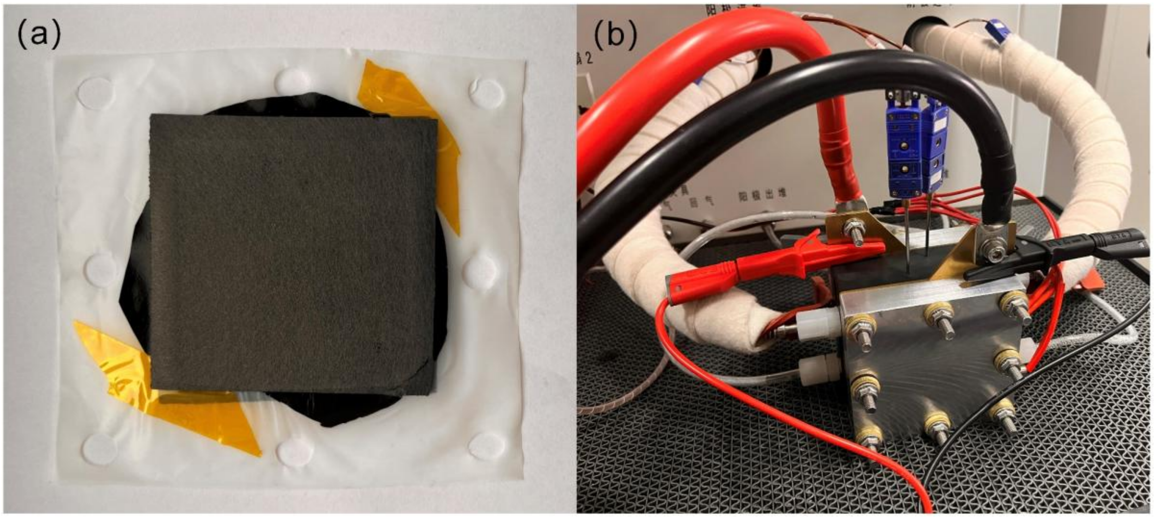

Figure S31. (a) Proton exchange membrane, (b) single battery for testing.

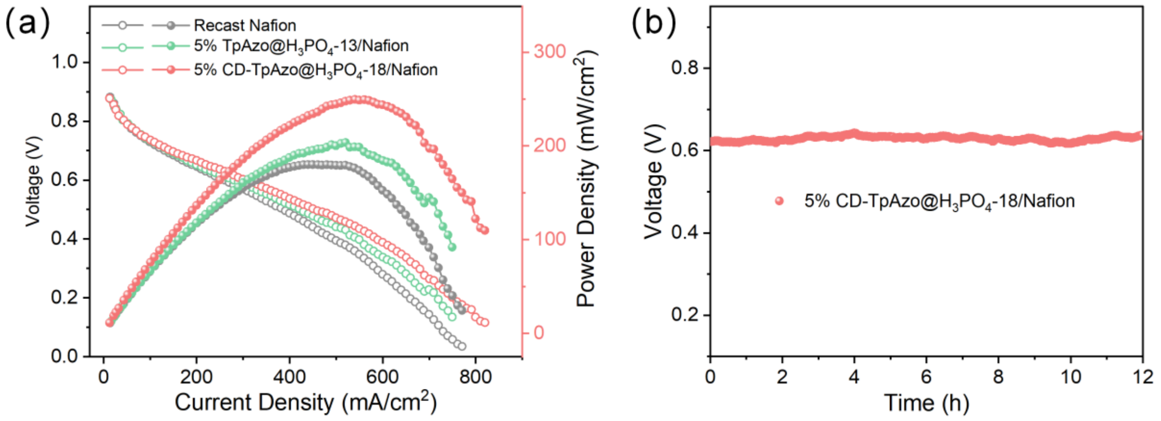

Figure S32. (a) Fuel cell polarization curves and power density curves measured at 70 °C and anhydrous condition using single H_2/O_2 cell assembly, (b) long term durability tests of the MEAs assembled with 5% CD-TpAzo@H_3PO_4-18/nafion membrane at 300 mA cm^{-2} under 70 °C and anhydrous condition.

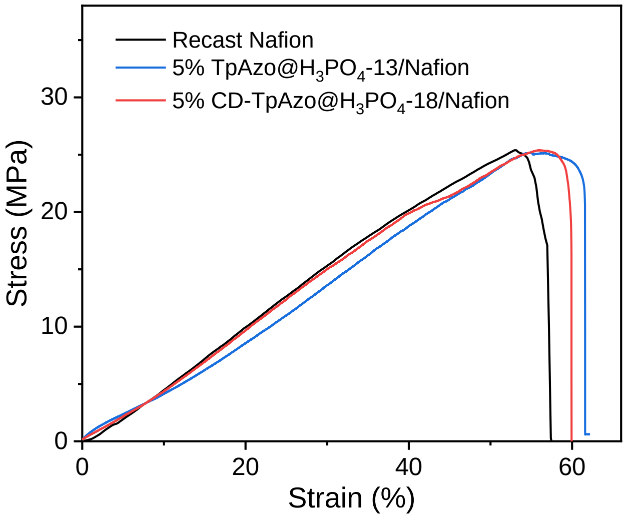

Figure S33. Stress-strain curves of Recast Nafion membrane, 5% TpAzo@H_3PO_4-13/nafion composite membranes and 5% CD-TpAzo@H_3PO_4-18/nafion composite membranes

Section S-7: Supporting tables

Table S1. Elemental analysis of COFs.

Sample		C [%]	H [%]	N [%]
TpAzo	Calcd	68.35	3.82	17.71
	Found	67.28	3.91	17.29
CD-TpAzo	Calcd	50.31	5.63	4.35
	Found	50.11	5.66	4.92

Table S2. Proton conductivity (S cm^{-1}) of the sample.

Conditions	TpAzo	TpAzo@H_3PO_4-5	TpAzo@H_3PO_4-10	TpAzo@H_3PO_4-13
70 °C	1.25×10^{-7}	9.43×10^{-7}	3.73×10^{-5}	1.29×10^{-3}
90 °C	1.39×10^{-7}	1.47×10^{-6}	5.44×10^{-5}	1.86×10^{-3}
110 °C	1.62×10^{-7}	3.11×10^{-6}	8.84×10^{-5}	2.55×10^{-3}
130 °C	1.93×10^{-7}	6.52×10^{-6}	1.77×10^{-4}	3.74×10^{-3}
150 °C	2.39×10^{-7}	1.41×10^{-5}	3.72×10^{-4}	5.74×10^{-3}

Table S3. Proton conductivity (S cm^{-1}) of the sample.

Conditions	CD-TpAzo	CD-TpAzo@H_3PO_4-5	CD-TpAzo@H_3PO_4-10	CD-TpAzo@H_3PO_4-15	CD-TpAzo@H_3PO_4-18
70 °C	1.65×10^{-7}	4.16×10^{-5}	5.24×10^{-4}	1.57×10^{-2}	0.26
90 °C	1.87×10^{-7}	5.89×10^{-5}	7.07×10^{-4}	2.02×10^{-2}	0.31
110 °C	2.15×10^{-7}	8.32×10^{-5}	1.01×10^{-3}	2.62×10^{-2}	0.41
130 °C	2.51×10^{-7}	1.57×10^{-4}	1.57×10^{-3}	3.72×10^{-2}	0.56
150 °C	3.15×10^{-7}	2.44×10^{-4}	2.83×10^{-3}	6.15×10^{-2}	0.78

Table S4. Single (H_2) gas permeance (Barrer, 1 Barrer = 3.35 $\times 10^{-16}$ mol m^{-1} s^{-1} Pa^{-1}) for Membrane at 25 ^oC.

**Membrane**	**H_2 permeance (Barrer)**
Recast nafion	8.06 $\pm$0.89
5% CD-TpAzo@H_3PO_4-18/Nafion	9.12 $\pm$0.85

Table S5. Comparison of proton conductivities in reported materials.

	Compound Name	Conductivity (S cm^{-1})	Conditions	References
1	EB-COF: PW_{12} [4]	3.32×10^{-3}	25 ^{o}C, 97% RH	*J. Am. Chem. Soc. 2016*
2	LiCl@RT-COF-1 [5]	6.45×10^{-3}	40 ^{o}C, 100% RH	*J. Am. Chem. Soc. 2017*
3	NUS-10@PVDF-50 [6]	1.58×10^{-2}	80 ^{o}C, 97% RH	*ACS Appl. Mater. Interfaces 2016*
4	H_3PO_4@NKCOF-10 [7]	6.97×10^{-2}	25 ^{o}C, 90% RH	*Nat. Commun. 2021*
5	ZUT-COF-SO_3H [8]	8.65×10^{-2}	80 ^{o}C, 98% RH	*J. Mater. Chem. A, 2023*
6	H_3PO_4@NKCOF-1 [9]	1.13×10^{-1}	80 ^{o}C, 98% RH	*Angew. Chem. Int. Ed. 2020*
7	IPC-COF [10]	3.80×10^{-1}	80 ^{o}C, 35% RH	*Adv. Mater. 2020*
8	SCOF [11]	5.40×10^{-1}	80 ^{o}C, 100% RH	*Angew. Chem. Int. Ed. 2021*
9	etidronic acid@COF-300 [12]	6.50×10^{-1}	90 ℃, 100% RH	*J. Mater. Chem. A, 2021*
10	TB-COF [13]	1.52×10^{-4}	120 ^{o}C, Anhydrous	*J. Mater. Chem. A, 2022*
11	phytic@TpPa-(SO_3H-Py) [14]	5.00×10^{-4}	120 ^{o}C, Anhydrous	*Chem. Mater. 2016*

12	CS/UiO-66(SO_3H)-6 [15]	1.52×10^{-3}	90 ^oC, Anhydrous	*ACS Appl. Mater. Interfaces 2018*
13	DHZIF-8/RN-3 [16]	3.66×10^{-3}	120 ^oC, Anhydrous	*Journal of Membrane Science 2022*
14	$[Zn(H_2PO_4)_2(C_2N_3H_3)_2]_n$ [17]	4.60×10^{-3}	150 ^oC, Anhydrous	*J. Am. Chem. Soc. 2016*
15	im@NKCOF-57-PA [18]	1.06×10^{-2}	160 ^oC, Anhydrous	*Small 2025*
16	H@TPT-COF [19]	1.27×10^{-2}	160 ^oC, Anhydrous	*Angew. Chem. Int. Ed. 2022*
17	TpAzo@H_3PO_4-13	1.27×10^{-2}	150 ^oC, anhydrous	This work
18	PA@TpDMAP/Pa-SO_3H [20]	1.56×10^{-2}	140 ^oC, Anhydrous	*Chem. Mater. 2025*
19	PA@EB-COF [21]	2.77×10^{-2}	180 ^oC, Anhydrous	*J. Mater. Chem. A, 2020*
20	COF-F6-H [22]	4.20×10^{-2}	140 ^oC, Anhydrous	*J. Am. Chem. Soc. 2020*
21	im@XJCOF-1 [23]	4.38×10^{-2}	140 ^oC, Anhydrous	*ACS Nano 2021*
22	LiBr@1 [24]	1.55×10^{-1}	110 ^oC, Anhydrous	*ACS Materials Lett. 2021*
23	TFPT-AF@H_3PO_4 [25]	3.90×10^{-1}	125 ^oC, Anhydrous	*Small 2024*
24	CD-TpAzo@H_3PO_4-18	7.80×10^{-1}	150 ^oC, Anhydrous	This work

Table S6. Fractional atomic coordinates for the unit cell of TpAzo.

Space group		P6/M	
Unit cell		a = b = 31.5 Å, c = 3.3 Å, α = β = 90º, γ = 120º	
Pawley refinement		R_p = 6.86%, R_{wp} = 4.32%	
atoms	x	y	z
O1	0.29695	0.57697	0
N2	0.38245	0.58505	0
C3	0.31382	0.61757	0
C4	0.39342	0.63145	0
C5	0.41468	0.5699	0
C6	0.46145	0.59947	0
C7	0.49111	0.58277	0
C8	0.47494	0.53664	0
C9	0.42853	0.50701	0
C10	0.39878	0.52354	0
C11	0.36359	0.64675	0
H12	0.42849	0.65656	0
H13	0.47589	0.63531	0
H14	0.34922	0.55946	0
H15	0.36308	0.50007	0
H16	0.52692	0.60571	0
H17	0.41558	0.4712	0
N1	0.50627	0.52081	1

Table S7. Fractional atomic coordinates (Portion) for the unit cell of CD-TpAzo.

Space group		P1	
Unit cell		a = b = 31.5 Å, c = 13.6 Å, α = β = 90º, γ = 120 º	
Pawley refinement		R_p = 5.31%, R_{wp} = 5.94%	
atoms	x	y	z
C1	0.35232	0.38069	0.57812
C2	0.37002	0.35183	0.52756
C3	0.4002	0.38241	0.45371
C4	0.37074	0.39942	0.40261
C5	0.3517	0.42582	0.45821
C6	0.319	0.44059	0.4125
O7	0.39956	0.34099	0.5822
O8	0.41288	0.35259	0.40353
O9	0.40443	0.43358	0.34272
O10	0.3243	0.3941	0.52625
O11	0.27851	0.39874	0.37323
C12	0.39153	0.42654	0.25719
C13	0.43107	0.42195	0.2099
C14	0.48002	0.46838	0.22222
C15	0.4756	0.51225	0.19642
C16	0.43248	0.51317	0.23749
C17	0.42351	0.55267	0.20052
O18	0.43493	0.38129	0.24026
O19	0.51771	0.46795	0.17224
O20	0.52017	0.55471	0.22242
O21	0.38829	0.46712	0.22567
O22	0.42289	0.55055	0.11115
C23	0.54807	0.59206	0.16327
C24	0.60023	0.60189	0.16881
C25	0.6194	0.61826	0.25756
C26	0.61347	0.66148	0.28355
C27	0.56174	0.6529	0.26593
C28	0.5589	0.6997	0.27453
O29	0.60243	0.55904	0.14496
O30	0.67037	0.63384	0.26235
O31	0.62208	0.66623	0.3716
O32	0.54706	0.63545	0.18131
O33	0.59828	0.74072	0.23342
C34	0.65182	0.71387	0.40541

C35	0.68763	0.71066	0.46454
C36	0.66113	0.67923	0.53971
C37	0.62931	0.69701	0.57964
C38	0.59403	0.69797	0.5149
C39	0.56409	0.7202	0.54854
O40	0.71499	0.69211	0.42162
O41	0.69598	0.68236	0.60043
O42	0.60142	0.66173	0.644
O43	0.62324	0.73023	0.4478
O44	0.52874	0.71476	0.48463
C45	0.59945	0.67856	0.72474
C46	0.61186	0.64968	0.78715
C47	0.57308	0.5954	0.78446
C48	0.52166	0.58845	0.78974
C49	0.5138	0.62202	0.73103
C50	0.46622	0.62242	0.74642
O51	0.65977	0.65707	0.77104
O52	0.58154	0.5707	0.85129
O53	0.4889	0.53841	0.76576
O54	0.55271	0.67182	0.74216
O55	0.45918	0.62935	0.83176
C56	0.44906	0.50753	0.81847
C57	0.44658	0.45763	0.82278
C58	0.43876	0.436	0.73546
C59	0.3934	0.43404	0.69734
C60	0.3936	0.48216	0.70214
C61	0.34411	0.47682	0.67834
O62	0.49097	0.46393	0.85635
O63	0.432	0.38793	0.7423
O64	0.39333	0.42205	0.61065
O65	0.40439	0.50082	0.78702
O66	0.30355	0.43372	0.71334
H67	0.32663	0.35966	0.62563
H68	0.33617	0.31599	0.50674
H69	0.42882	0.41781	0.47632
H70	0.33874	0.36832	0.36709
H71	0.38305	0.45984	0.48712
H72	0.3399	0.46696	0.36151
H73	0.30981	0.46028	0.45794
H74	0.40759	0.32223	0.57019

H75	0.42551	0.36807	0.34859
H76	0.25925	0.39071	0.4061
H77	0.35472	0.39354	0.24938
H78	0.42267	0.41827	0.14198
H79	0.49006	0.47033	0.28893
H80	0.47233	0.51086	0.12895
H81	0.43929	0.51789	0.30811
H82	0.45445	0.59056	0.22219
H83	0.38655	0.54439	0.22488
H84	0.41116	0.36294	0.20258
H85	0.50546	0.45645	0.10387
H86	0.38296	0.51888	0.08202
H87	0.53042	0.57926	0.10619
H88	0.62275	0.63086	0.12917
H89	0.60126	0.58792	0.30257
H90	0.63997	0.69356	0.24988
H91	0.53334	0.62573	0.31169
H92	0.55892	0.70676	0.33738
H93	0.52272	0.69256	0.24927
H94	0.5859	0.54091	0.19173
H95	0.67292	0.60597	0.27176
H96	0.59307	0.74206	0.18353
H97	0.6677	0.74071	0.3576
H98	0.71473	0.74651	0.48483
H99	0.63649	0.64086	0.52264
H100	0.65207	0.7339	0.60687
H101	0.57244	0.6622	0.49156
H102	0.53252	0.68665	0.59799
H103	0.58557	0.75876	0.56518
H104	0.70026	0.67045	0.38832
H105	0.62397	0.71928	0.72352
H106	0.61317	0.6633	0.85117
H107	0.57491	0.57896	0.72333
H108	0.51339	0.59453	0.85516
H109	0.51651	0.6129	0.66354
H110	0.46464	0.64856	0.70376
H111	0.43805	0.59127	0.72321
H112	0.66463	0.65993	0.71112
H113	0.4676	0.65793	0.86209
H114	0.45327	0.52517	0.87881

H115	0.41482	0.4322	0.86508
H116	0.47019	0.45859	0.69381
H117	0.36309	0.40716	0.7304
H118	0.42095	0.50589	0.65464
H119	0.34075	0.50802	0.69868
H120	0.34215	0.47672	0.60796
H121	0.50091	0.43727	0.87212
H122	0.43021	0.37749	0.67667
H123	0.31683	0.44095	0.78069
O124	0.12919	0.77139	-0.00187
N125	0.21469	0.77947	-0.00187
C126	0.14606	0.81199	-0.00187
C127	0.22566	0.82587	-0.00187
C128	0.24692	0.76432	-0.00187
C129	0.29369	0.79389	-0.00187
C130	0.32335	0.77719	-0.00187
C131	0.30718	0.73106	-0.00187
C132	0.26077	0.70143	-0.00187
C133	0.23102	0.71796	-0.00187
C134	0.19583	0.84117	-0.00187
H135	0.26073	0.85098	-0.00187
H136	0.30813	0.82973	-0.00187
H137	0.18146	0.75388	-0.00187
H138	0.19532	0.69449	-0.00187
H139	0.35916	0.80013	-0.00187
H140	0.24782	0.66562	-0.00187
O141	0.25527	0.9144	-0.00187
N142	0.24719	0.99182	-0.00187
C143	0.21467	0.89067	-0.00187
C144	0.20079	0.95639	-0.00187
C145	0.26234	1.0392	-0.00187
C146	0.23277	1.0564	-0.00187
C147	0.24947	1.10276	-0.00187
C148	0.2956	1.13272	-0.00187
C149	0.32523	1.11594	-0.00187
C150	0.3087	1.06966	-0.00187
C151	0.18549	0.91126	-0.00187
H152	0.17568	0.96635	-0.00187
H153	0.19693	1.035	-0.00187
H154	0.27278	0.98418	-0.00187

H155	0.33217	1.05743	-0.00187
H156	0.22653	1.11563	-0.00187
H157	0.36104	1.1388	-0.00187
O158	0.11226	0.89747	-0.00187
N159	0.03484	0.81197	-0.00187
C160	0.13599	0.8806	-0.00187
C161	0.07027	0.801	-0.00187
C162	-0.01254	0.77974	-0.00187
C163	-0.02974	0.73297	-0.00187
C164	-0.0761	0.70331	-0.00187
C165	-0.10606	0.71948	-0.00187
C166	-0.08928	0.76589	-0.00187
C167	-0.043	0.79564	-0.00187
C168	0.1154	0.83083	-0.00187
H169	0.06031	0.76593	-0.00187
H170	-0.00834	0.71853	-0.00187
H171	0.04248	0.8452	-0.00187
H172	-0.03077	0.83134	-0.00187
H173	-0.08897	0.6675	-0.00187
H174	-0.11214	0.77884	-0.00187
O175	0.53529	0.61745	-0.00187
N176	0.44979	0.60937	-0.00187
C177	0.51842	0.57685	-0.00187
C178	0.43882	0.56297	-0.00187
C179	0.41756	0.62452	-0.00187
C180	0.37079	0.59495	-0.00187
C181	0.34113	0.61165	-0.00187
C182	0.3573	0.65778	-0.00187
C183	0.40371	0.68741	-0.00187
C184	0.43346	0.67088	-0.00187
C185	0.46865	0.54767	-0.00187
H186	0.40375	0.53786	-0.00187
H187	0.35635	0.55911	-0.00187
H188	0.48302	0.63496	-0.00187
H189	0.46916	0.69435	-0.00187
H190	0.30532	0.58871	-0.00187
H191	0.41666	0.72322	-0.00187
O192	0.40921	0.47444	-0.00187
N193	0.41729	0.39702	-0.00187
C194	0.44981	0.49817	-0.00187

C195	0.46369	0.43245	-0.00187
C196	0.40214	0.34964	-0.00187
C197	0.43171	0.33244	-0.00187
C198	0.41501	0.28608	-0.00187
C199	0.36888	0.25612	-0.00187
C200	0.33925	0.2729	-0.00187
C201	0.35578	0.31918	-0.00187
C202	0.47899	0.47758	-0.00187
H203	0.4888	0.42249	-0.00187
H204	0.46755	0.35384	-0.00187
H205	0.3917	0.40466	-0.00187
H206	0.33231	0.33141	-0.00187
H207	0.43795	0.27321	-0.00187
H208	0.30344	0.25004	-0.00187
O209	0.55222	0.49137	-0.00187
N210	0.62964	0.57687	-0.00187
H204	0.52849	0.50824	-0.00187
H205	0.59421	0.58784	-0.00187
H206	0.67702	0.6091	-0.00187
H207	0.69422	0.65587	-0.00187
H208	0.74058	0.68553	-0.00187
O209	0.77054	0.66936	-0.00187
N210	0.75376	0.62295	-0.00187

Section S-8: Supporting references

1. Park J, Koh J. The synthesis and spectral properties of an encapsulated aminoazobenzene dye. *Dyes Pigm.* 2009; **82**: 347-352.

2. Chandra S, Kundu T, Kandambeth S et al. Phosphoric acid loaded azo (-N = N-) based covalent organic framework for proton conduction. *J. Am. Chem. Soc.* 2014; **136**: 6570-6573.

3. Yang Q, Li X, Xie C et al. Expanding the dimensionality of proton conduction enables ultrahigh anhydrous proton conductivity of phosphoric acid-doped covalent-organic frameworks. *Nano Res.* 2023; **16**: 10946-10955.

4. Ma H, Liu B, Li B et al. Cationic Covalent Organic Frameworks: A Simple Platform of Anionic Exchange for Porosity Tuning and Proton Conduction. *J. Am. Chem. Soc.* 2016; **138**: 5897-5903.

5. Montoro C, Rodriguez-San-Miguel D, Polo E et al. Ionic Conductivity and Potential Application for Fuel Cell of a Modified Imine-Based Covalent Organic Framework. *J. Am. Chem. Soc.* 2017; **139**: 10079-10086.

6. Peng Y, Xu G, Hu Z et al. Mechanoassisted Synthesis of Sulfonated Covalent Organic Frameworks with High Intrinsic Proton Conductivity. *ACS Appl. Mater. Interfaces* 2016; **8**: 18505-18512.

7. Wang Z, Yang Y, Zhao Z et al. Green synthesis of olefin-linked covalent organic frameworks for hydrogen fuel cell applications. *Nat. Commun.* 2021; **12**: 1982.

8. Shao Z, Xue X, Gao K et al. Sulfonated covalent organic framework packed Nafion membrane with high proton conductivity for H_2/O_2 fuel cell applications. *J. Mater. Chem. A* 2023; **11**: 3446-3453.

9. Yang Y, He X, Zhang P et al. Combined Intrinsic and Extrinsic Proton Conduction in Robust Covalent Organic Frameworks for Hydrogen Fuel Cell Applications. *Angew.Chem. Int.Ed.* 2020; **59**: 3678-3684.

10. Cao L, Wu H, Cao Y et al. Weakly Humidity-Dependent Proton-Conducting COF Membranes. *Adv. Mater.* 2020; **32**: 2005565.

11. Liu L, Yin L, Cheng D et al. Surface-Mediated Construction of an Ultrathin Free-Standing Covalent Organic Framework Membrane for Efficient Proton Conduction. *Angew. Chem. Int. Ed.* 2021; **60**: 14875-14880.

12. Fan C, Geng H, Wu H et al. Three-dimensional covalent organic framework membrane for efficient proton conduction. *J. Mater. Chem. A* 2021; **9**: 17720-17723.

13. Guo Y, Zou X, Li W et al. High-density sulfonic acid-grafted covalent organic frameworks with efficient anhydrous proton conduction. *J. Mater. Chem. A* 2022; **10**: 6499-6507.

14. Chandra S, Kundu T, Dey K et al. Interplaying Intrinsic and Extrinsic Proton Conductivities in Covalent Organic Frameworks. *Chem. Mater.* 2016; **28**: 1489-1494.

15. Dong X-Y, Wang J-H, Liu S-S et al. Synergy between Isomorphous Acid and Basic Metal–Organic Frameworks for Anhydrous Proton Conduction of Low-Cost Hybrid Membranes at High Temperatures. *ACS Appl. Mater. Interfaces* 2018; **10**: 38209-38216.

16. Rao Z, Lan M, Wang Z et al. Effectively facilitating the proton conduction of proton exchange membrane by polydopamine modified hollow metal-organic framework. *Journal of Membrane Science* 2022; **644**: 1-9.

17. Inukai M, Horike S, Itakura T et al. Encapsulating Mobile Proton Carriers into Structural Defects in Coordination Polymer Crystals: High Anhydrous Proton Conduction and Fuel Cell Application. *J. Am. Chem. Soc.* 2016; **138**: 8505-8511.

18. Hao L, Lin E, Liu J et al. Skeleton Regulation of Covalent-Organic Frameworks From 2D to 3D Networks for High Anhydrous Proton Conduction. *Small* 2025; **21**: 2411954.

19. Jiang G, Zou W, Ou Z et al. Tuning the Interlayer Interactions of 2D Covalent Organic Frameworks Enables an Ultrastable Platform for Anhydrous Proton Transport. *Angew. Chem. Int. Ed.* 2022; **61**: e202208086.

20. Joseph V, Maegawa K, Wlazło M et al. Dual-Acid-Tailored Ionic Covalent Organic Frameworks for High-Temperature Proton Conduction under Anhydrous Conditions and the Practical Opportunities. *Chem. Mater.* 2025; **37**: 2561-2568.

21. Chen S, Wu Y, Zhang Y et al. Tuning proton dissociation energy in proton carrier doped 2D covalent organic frameworks for anhydrous proton conduction at elevated temperature. *J. Mater. Chem. A* 2020; **8**: 13702-13709.

22. Wu X, Hong YL, Xu B et al. Perfluoroalkyl-Functionalized Covalent Organic Frameworks with Superhydrophobicity for Anhydrous Proton Conduction. *J. Am. Chem. Soc.* 2020; **142**: 14357-14364.

23. Fu Y, Wu Y, Chen S et al. Zwitterionic Covalent Organic Frameworks: Attractive Porous Host for Gas Separation and Anhydrous Proton Conduction. *ACS Nano* 2021; **15**: 19743-19755.

24. Zhang K, Wen G-H, Yang X-J et al. Anhydrous Superprotonic Conductivity of a Uranyl-Based MOF from Ambient Temperature to 110 ℃. *ACS Materials Lett.* 2021; **3**: 744-751.

25. Jiang S, Niu H, Gu X et al. Perfluoroalkyl Functionalized Superhydrophobic Covalent Organic Frameworks for Excellent Oil-Water Membrane Separation and Anhydrous Proton Conduction. *Small* 2024; **20**: 2403772.
